# Gradient-Layered MXene/Hollow Lignin Nanospheres Architecture Design for Flexible and Stretchable Supercapacitors

**DOI:** 10.1007/s40820-024-01512-3

**Published:** 2024-10-17

**Authors:** Haonan Zhang, Cheng Hao, Tongtong Fu, Dian Yu, Jane Howe, Kaiwen Chen, Ning Yan, Hao Ren, Huamin Zhai

**Affiliations:** 1 Jiangsu Provincial Key Lab of Sustainable Pulp and Paper Technology and Biomass Materials, NanJing Forestry University, Nanjing, 210037 People’s Republic of China; 2 Department of Chemical Engineering and Applied Chemistry, University of Toronto, 200 College Street, Toronto, ON M5S 3E5 Canada; 3 Department of Materials Science and Engineering, University of Toronto, 184 College Street, Toronto, ON M5S 3E4 Canada; 4 College of Materials Science and Engineering, Nanjing Forestry University, Nanjing, 210037 People’s Republic of China

**Keywords:** Hollow lignin nanospheres, MXene, Gradient-layered architecture, Wrinkled electrodes, Stretchable supercapacitors

## Abstract

With the rapid development of flexible wearable electronics, the demand for stretchable energy storage devices has surged. In this work, a novel gradient-layered architecture was design based on single-pore hollow lignin nanospheres (HLNPs)-intercalated two-dimensional transition metal carbide (Ti_3_C_2_T_x_ MXene) for fabricating highly stretchable and durable supercapacitors. By depositing and inserting HLNPs in the MXene layers with a bottom-up decreasing gradient, a multilayered porous MXene structure with smooth ion channels was constructed by reducing the overstacking of MXene lamella. Moreover, the micro-chamber architecture of thin-walled lignin nanospheres effectively extended the contact area between lignin and MXene to improve ion and electron accessibility, thus better utilizing the pseudocapacitive property of lignin. All these strategies effectively enhanced the capacitive performance of the electrodes. In addition, HLNPs, which acted as a protective phase for MXene layer, enhanced mechanical properties of the wrinkled stretchable electrodes by releasing stress through slip and deformation during the stretch-release cycling and greatly improved the structural integrity and capacitive stability of the electrodes. Flexible electrodes and symmetric flexible all-solid-state supercapacitors capable of enduring 600% uniaxial tensile strain were developed with high specific capacitances of 1273 mF cm^−2^ (241 F g^−1^) and 514 mF cm^−2^ (95 F g^−1^), respectively. Moreover, their capacitances were well preserved after 1000 times of 600% stretch-release cycling. This study showcased new possibilities of incorporating biobased lignin nanospheres in energy storage devices to fabricate stretchable devices leveraging synergies among various two-dimensional nanomaterials.

**Supplementary Information:**

The online version contains supplementary material available at 10.1007/s40820-024-01512-3.

## Highlights

- A novel gradient-layered architecture based on single-pore hollow lignin nanospheres (HLNPs)-intercalated MXene layers was created to fabricate highly stretchable (600%) and durable (1000 cycling) supercapacitor electrodes.
- The architecture reduced the overstacking of MXene, and the micro-chamber structure of HLNPs better utilized lignin’s pseudocapacitive property to improve ion and electron accessibility (specific capacitance reached 1273 mF cm^−2^).
- HLNPs enhanced mechanical durability and capacitive stability of the integrated wrinkled electrodes during the stretch-release cycling.

**Supplementary Information:**

The online version contains supplementary material available at 10.1007/s40820-024-01512-3.

## Graphical abstract

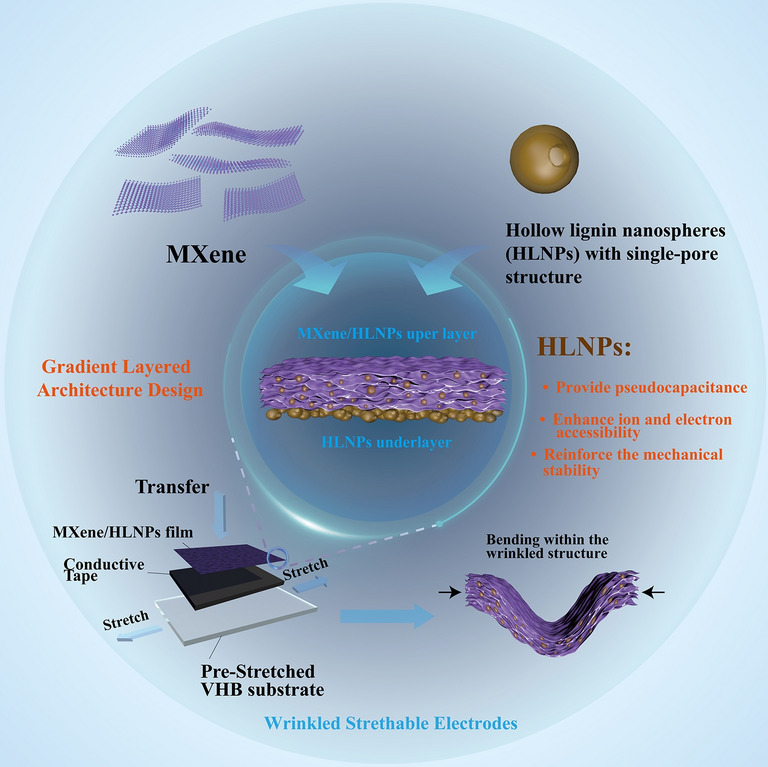

## Introduction

In recent years, flexible electronics have experienced rapid development in various fields such as wearable multifunctional sensor [1–3], electronic skin [4], human–machine interface [5], soft robot [6], and flexible display. This advancement has led to an increasing interest in developing energy storage devices that are not only flexible but also mechanically compatible with these emerging devices. Among various options, stretchable supercapacitors are seen as the ideal candidates for powering flexible electronics due to their fast charging/discharging process, excellent cycling stability and easy fabrication procedure [7, 8].

MXene is regarded as a promising electrode material for flexible electrochemical energy storage devices owing to its desirable properties, such as metal-like electronic conductivity (exceeding 10,000 S cm^−1^), solution processability, and high volumetric capacitance (up to 1500 F cm^−3^) [9–14]. Few-layer MXene nanosheets after well dispersed in solution were able to easily assemble into flexible films through vacuum filtration as supercapacitor electrodes [15]. The intrinsic layer structure of parallel stacked MXene nanosheet formed flexible MXene films that were able to withstand bending. But these films were prone to fracture during tensile deformation and could not be used directly in stretchable devices [16–18]. An interesting strategy to address this limitation was to build the wrinkled structure of the MXene film by adhering the MXene film to a pre-stretched elastomer, followed by strain release. The wrinkled structure provided reserved space for the subsequent tensile deformation [19–22]. This clever structural design leveraged MXene film's bending resilience while effectively circumventing its actual strain in the parallel direction of the MXene layer, providing the electrodes with good stretchability and electrochemical stability [23]. Nonetheless, the mechanical strength of the pure MXene film was not sufficient to withstand the compressive effect during strain recovery after transferring to a larger-scale pre-stretched substrate, which limited the ultimate tensile strain and electrochemical stability of the electrodes [19]. The incorporation of additives into MXene composite films can improve the mechanical properties of MXene electrodes [18, 24]. However, the outcome of using this strategy was double fold. On one hand, the rapid increase in material resistance during stretching led to unstable electrochemical properties of the energy storage devices. On the other hand, the doped components with poor capacitance and conductivity also reduced the loading of the electrode active material and decreased the specific capacitance of the electrode. Furthermore, similar to other 2D materials, the re-stacking of MXene during practical utilization reduced the effective contact area between the electrolyte and the active sites on the electrode surface, leading to an obvious gap between the actual capacitance and the theoretical value [25].

The integration of intercalating substances into the MXene nanosheet layers was shown to help prevent over-stacking of the MXene layers [26–29]. However, this often involved the introduction of normally inactive substances, which reduced the specific capacitance of the electrode (when normalized against the mass of the overall electrode materials). Meanwhile, lignin, as one of the main components of the lignocellulosic biomass cell wall and the only non-petroleum resource of renewable aromatic compounds available in nature, emerged as a potential solution [30, 31]. The phenolic hydroxyl structure, which is abundant in lignin, can be converted into a redox-active hydrazine/hydroquinone (Q/QH_2_) structure. The phenolic hydroxyl structure, consisting of 6 carbon and 2 oxygen atoms, can store two electrons and protons, giving it a high theoretical specific capacitance in the positive potential range (with an electron charge density of 2 faradays/108 g, i.e., 1787 C g^−1^) [32–36]. The abundant source, low price, good biocompatibility, environmental friendliness, and high pseudocapacitance of lignin make it an ideal candidate for the fabrication of flexible wearable energy storage devices. However, pure lignin is virtually electrically insulating, which severely hinders its application in energy storage applications. Lignin must be combined with other materials with high electronic and ionic conductivities by suitable means to fully utilize its theoretical specific capacitance [37].

In this study, we report a novel gradient electrode design [38] to hierarchically intercalate single-pore hollow lignin nanospheres (HLNPs) into MXene nanosheet layers, thereby constructing a layered porous structure within the electrode film with the density of MXene layer stacks decreasing in a top-to-bottom gradient. Then, the stretchable and high-pseudocapacitance supercapacitor was fabricated by transferring the composite film to a pre-stretched flexible substrate. The intercalation of thin-walled single-hole hollow lignin nanospheres served to enlarge the interlayer spacing of MXene, enhancing ion accessibility. Simultaneously, the close contact between the high specific surface area of lignin nanosphere walls and the MXene lamellae facilitated the full utilization of lignin pseudocapacitance. Additionally, the density gradient in the electrode film improved its structural integrity during the stretch-release process. The resulting flexible electrodes and assembled all-solid-state symmetric supercapacitors exhibited remarkable stretching properties, enduring a uniaxial tensile strain of up to 600%. They also demonstrated high specific capacitances of 1273 mF cm^−2^ (241 F g^−1^) and 514 mF cm^−2^ (95 F g^−1^), respectively. The capacitance retention of the supercapacitor after 10,000 charge/discharge cycles was 82%, and the supercapacitor maintained excellent electrochemical stability under various strain levels. This design strategy combined stretchability with high pseudocapacitance to the flexible electrode, paving the way for broadening the practical application of various 2D nanomaterials in flexible electronics for developing next-generation high-performance energy storage devices.

## Experimental Section

### Materials

Lithium fluoride (LiF, ≥ 99.98% trace metals basis), hydrochloric acid (HCl, made using ACS reagent 37 wt%), Ti_3_AlC_2_ (Particle size < 40 μm), tetrahydrofuran (THF, ACS reagent, ≥ 99%), polyvinyl alcohol (PVA, average Mw 130,000, ≥ 99% hydrolyzed), and sulfuric acid (H_2_SO_4_, 95%–98%) were purchased from Sigma-Aldrich Inc. Kraft lignin was purchased from UPM (BioPiva^TM^190, dried, softwood Kraft lignin with Mw of ~ 3000 g mol^−1^). The acrylic elastomer substrates were obtained from 3 M Inc. (VHB 4910). Carbon conductive tapes (16,073) were purchased from Ted Pella Inc.

### Methods

#### ***Synthesis of Ti***_***3***_***C***_***2***_***T***_***x***_*** MXene***

The Ti_3_C_2_T_x_ MXene was synthesized using a modified version of the minimally intensive layer delamination (MILD) method described in previous literature [12]. Briefly, 1.6 g of LiF was dissolved in 20 mL of 9 M HCl in a poly(tetrafluoroethylene) flask and stirred at 40 °C for 30 min until completely dissolved. Subsequently, 1 g of Ti_3_AlC_2_ was slowly added, and the reaction proceeded for 48 h. The resulting slurry was centrifuged at 4000 rpm and washed with deionized water until reaching a pH > 6. The precipitate was then dispersed in water, purged with N_2_, and sonicated for 30 min in an ice bath. The suspension was centrifuged again at 4000 rpm for 30 min, and the supernatant was collected. The resulting monolayer of MXene suspension was stored under a nitrogen atmosphere at 5 °C. The concentration of the obtained Ti_3_C_2_T_x_ MXene suspension was 17 mg mL^−1^.

#### Preparation of Hollow Lignin Nanospheres

Hollow lignin nanospheres were synthesized by dissolving Kraft lignin in THF, sonicated in an ultrasonic cleaner (Branson 5210, 140 W) for 5 min, and filtered with a 0.45 μm cartridge to ensure complete dissolution of lignin. 5 mL of lignin/THF solution with a concentration of 1.5 mg mL^−1^ was added to a beaker. Subsequently, 45 mL of deionized water was added dropwise to the lignin/THF solution using a peristaltic pump at a rate of 2 mL min^−1^, while stirring continuously at 600 rpm with a magnetic bar. The resulting dispersion was then transferred into a dialysis bag with a molecular weight cut-off of 500 Da and dialyzed for 72 h with the deionized water being continuously renewed to remove the THF [39]. The obtained dispersion was then centrifuged at 2500, 5000, and 7500 rpm for collecting the precipitate and supernatant, respectively, to obtain size-graded HLNPs. The concentration of all size-graded HLNPs samples was adjusted to about 0.1 mg mL^−1^ by adding deionized water.

#### Preparation of the Hierarchically Intercalated MXene/HLNPs Composite Film

10 mL of 0.1 mg mL^−1^ HLNPs suspension was first filtered through a PVDF membrane (0.22 μm) via vacuum-assisted filtration to build the substructure of the composite film. Then, 1 mL of Ti_3_C_2_T_x_ MXene dispersion and 15 mL of HLNPs suspension were mixed and shaken well for 10 min before being poured into the filtration flask. The cascading sedimentation structure of HLNPs and MXene was constructed during the slow filtration process. The obtained composite film was easily peeled off from the membrane after drying in a vacuum oven at 40 °C for 3 h. The thickness of the prepared electrode film is about 5 μm.

#### Fabrication of the Stretchable Electrodes

The MXene/HLNPs film was first firmly laminated to a thin conductive tape (125 µm in thickness) with the HLNPs substrate as the contact layer. The conductive tape layer was then transferred onto a 600% uniaxial pre-stretched acrylic elastomer substrate. By releasing the pre-stretched elastic substrate, stretchable electrodes with wrinkled structures were obtained due to the strong adhesion between the tape and the film.

#### Characterization Methods

Scanning electron microscopy (SEM) and energy-dispersive X-ray spectroscopy (EDS) were carried out in a SU7000 (Hitachi, Japan) with an Ultim Max 80mm2 X-ray spectrometer (Oxford Instruments, United Kingdom) and a QUANTA FEG 250 (FEI, USA). Bright-field scanning transmission electron microscopy (BF-STEM) was conducted in the HT7700 (Hitachi, Japan) and the SU7000 using a dedicated sample holder (Hitachi, Japan) [40]. Fourier transform infrared spectra (FTIR) were collected on an iS50 spectrometer (Thermo Scientific, USA) in attenuated total reflectance mode, scanned 32 times from 450 to 4000 cm^−1^ with a resolution of 4 cm^−1^. Raman analyses were carried out at room temperature with a 532 nm laser excitation, using a SENTERRA dispersive Raman microscope (Bruker, USA) with a laser power of 10 mW. X-ray diffraction (XRD) patterns of the nanosheets were collected by a diffractometer (PW1830, 40 kV, 40 mA, Philips) with a Cu Kα (*λ* = 0.154 nm) Ni filter, collected from 5° to 50° with an increment of 0.02°/1.5 s. X-ray photoelectron (XPS) spectroscopy was performed on K-Alpha XPS spectrometer (Thermo Fisher Scientific, E. Grinstead, UK) with a monochromatic Al Kα X-rays of 400 µm nominal spot size. The tensile tests were performed on a universal testing machine (Instron 5965, USA) equipped with a 5 kN load cell. Surface charge properties of samples were investigated via zeta potential analysis (Zeta Plus, Brookhaven Instrument Corporation, USA). Brunauer–Emmett–Teller (BET) method was adopted for the analysis of specific surface area. Samples were degassed at 120 °C for 24 h under vacuum prior to the test. The test was conducted in NOVA 1200e analyzer (Quantachrome, USA) using N_2_ as absorbate at 77.35 K. Surface morphology of single nanosheet of MXene was captured by atomic force microscope (AFM, Nanosurf, CoreAFM, Switzerland).

#### Electrochemical Measurements

The electrochemical performance of stretchable electrodes was assessed in a 1 M H_2_SO_4_ aqueous electrolyte using an electrochemical workstation (Corrtest CS310M). The evaluation was conducted using a three-electrode cell, with Ag/AgCl in saturated KCl as the reference electrode and a Pt mesh as the counter electrode. A potential window of − 0.2–0.4 V was chosen for cyclic voltammetry (CV) and galvanostatic charge/discharge (GCD) measurements. Electrochemical impedance spectroscopy (EIS) was performed over a frequency range from 10^–2^ to 10^6^ Hz with an amplitude of 10 mV at the open circuit potential.

#### Fabrication of Stretchable Symmetric Supercapacitors

An all-solid-state stretchable symmetric supercapacitor was assembled by two as-prepared stretchable multilayered electrodes and a layer of PVA hydrogel (prepared by dissolving 2.5 g PVA into 25 mL of 1 M H_2_SO_4_) as both the electrolyte and the separator. Conductive copper tapes were used as current collectors by attaching them to the edges of the MXene/HLNPs films.

## Results and Discussion

### Fabrication and Characterization of the Stretchable Electrodes

**Fig. 1:**
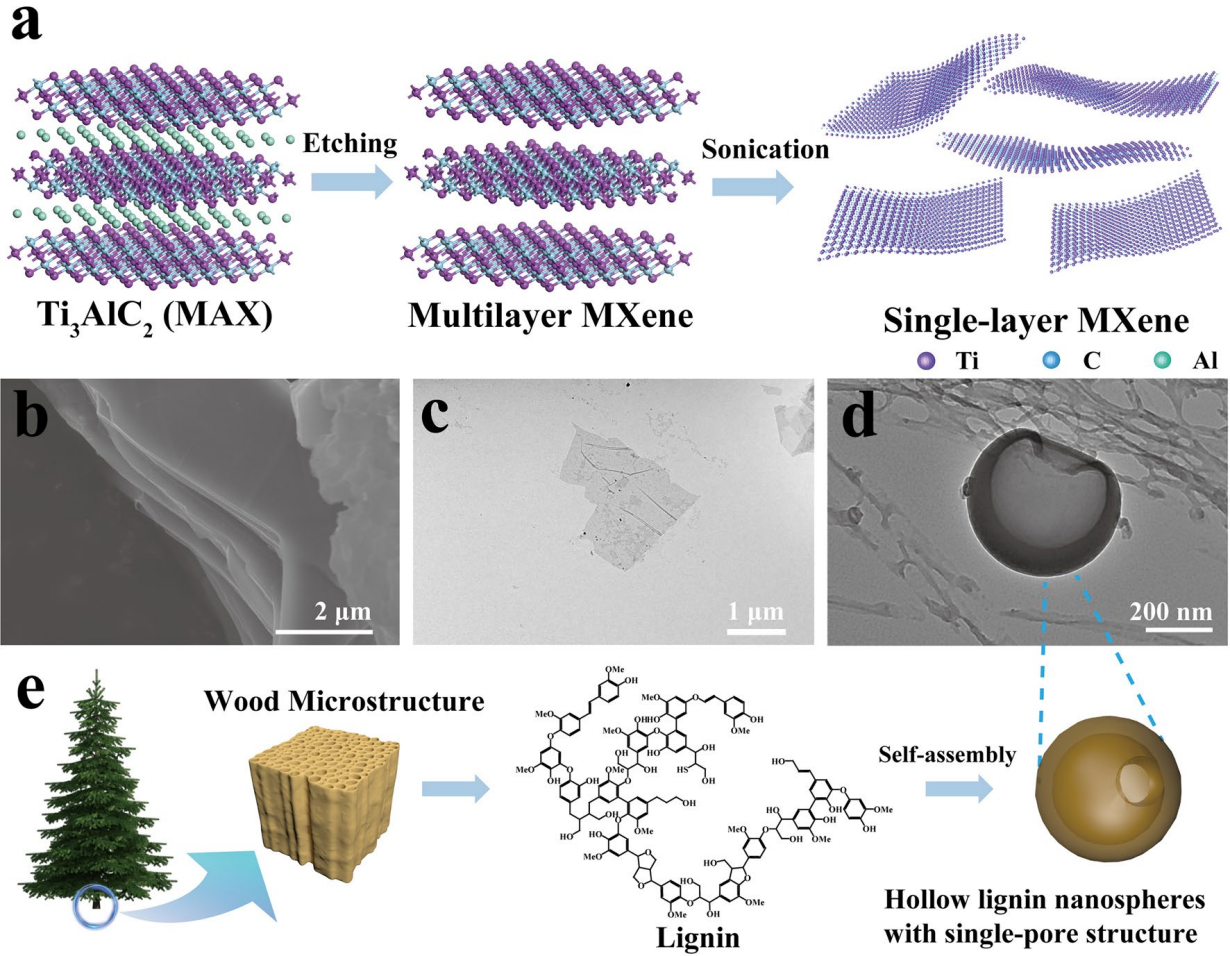
**a** Schematic illustration of MXene synthesis. **b** SEM image of multilayered MXene. **c** TEM image of monolayered MXene. **d** TEM image of the assembled HLNPs. **e** Schematic illustration of HLNPs synthesis

**Fig. 2:**
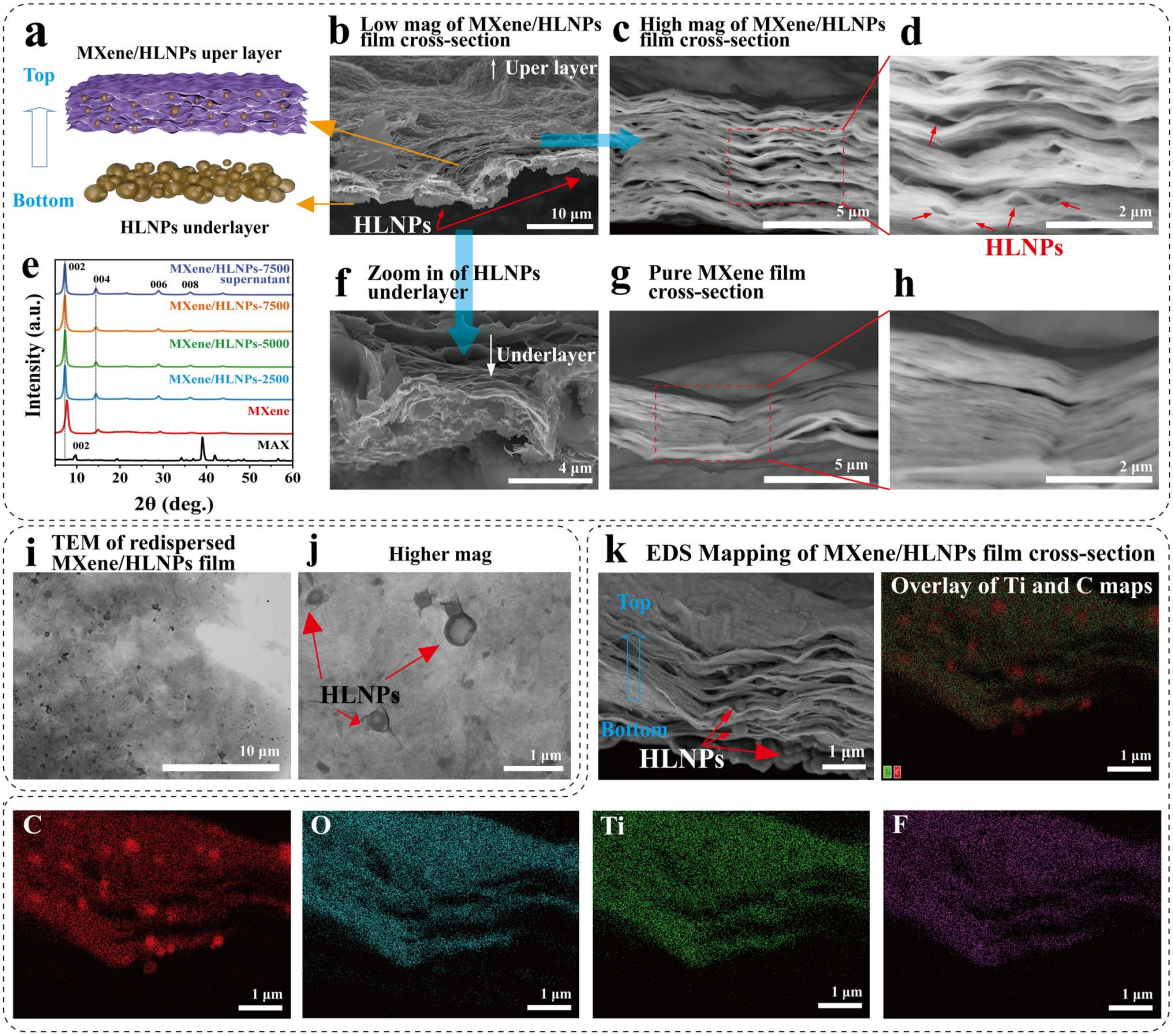
**a** Schematic diagram of the structure of MXene/HLNPs film. **b–d** SEM image of MXene/HLNPs film cross section of different magnifications. **e** XRD of MAX, MXene film and MXene film with different HLNPs. **f** Zoom in of HLNPs underlayer section of MXene/HLNPs film observed by SEM. **g, h** SEM images of pure MXene film cross section. **i, j** TEM images of redispersed MXene/HLNPs film. **k** SEM–EDS mapping of the cross section of MXene/HLNPs film

Flexible and stretchable MXene/HLNPs composite film electrodes, featuring intricate hierarchical structures, were constructed. This was achieved through a novel fabrication process that synergistically combined hierarchical sedimentation and vacuum-assisted filtration methods. Subsequently, a precise dry-transferring technique was employed to finalize the electrode assembly. Specific manufacturing approaches and discussions are as follows:

Firstly, Ti_3_C_2_T_*x*_ MXene nanosheets were synthesized by etching Ti_3_AlC_2_ MAX phase powders through MILD method (in situ hydrofluoric acid, as illustrated in Fig. 1a). As shown in Fig. 1b, the SEM image reveals the multilayered morphology of the etched Ti_3_C_2_T_*x*_, which suggests the removal of Al atoms and successful exfoliation. After further treatment by ultrasonication and centrifugation, the few-layer and single-layer structures of MXene were dispersed in water homogeneously, which were observed in the TEM images (Fig. 1c). The planar size of as-prepared MXene nanosheets retained a large planar size of 1–5 μm. The larger size of the monolayer MXene nanosheet was preserved as much as possible during preparation by multiple centrifugal expansions, an increased hand-shaking time, and a shorter sonication time. The average thickness of the MXene nanosheets measured by atomic force microscopy (AFM) was 1.86 nm, demonstrating the successful preparation of monolayer MXene (Fig. S1). XRD analysis also suggested the entire MAX phase was fully transformed into MXene in the obtained MXene dispersion (Fig. 2e). The dominant peak at 2*θ* = 39.00° (characteristic of Ti_3_AlC_2_) disappeared, indicating Al removal, while the diffraction peak of MXene (002) shifted to a lower position, indicating the structural expansion caused by Al substitution [15].

The hollow lignin nanospheres with the single-pore structure were easily achieved by solvent exchange method [39]. Briefly, by dropwise addition of the counter-solvent (deionized water) to the lignin/THF solution, the hollow lignin nanospheres were formed via layer-by-layer self-assembly from outside to inside based on π–π interactions [39]. Morphology of the obtained HLNPs is shown in Fig. 1d, and the schematic illustration of HLNPs synthesis is shown in Fig. 1e. The size of the hollow lignin nanospheres was further graded and screened by centrifugation with varying speeds and the characteristics were confirmed by TEM (Fig. S2). The diameter distribution of HLNPs was between 100 and 500 nm, and the diameter of the precipitated HLNPs tended to decrease with an increasing centrifugation speed. A small number of hemispheres and inter-wrapped lamellar structures were present in the supernatant from centrifugation up to 7500 rpm, which was attributed to the incomplete self-assembly of a portion of lignin. Unless specifically noted, all HLNPs used for the subsequent HLNPs/MXene composite film preparation were taken from HLNPs fractions precipitated by centrifugation between 2500 and 7500 rpm to remove size extremes.

The composite film with a hierarchical architecture was fabricated employing a two-step process that leveraged both the vacuum-assisted filtration and the graded sedimentation. Initially, the uneven underlying layer composed of HLNPs was formed. Then, over this HLNPs substrate layer, a mixed suspension of MXene/HLNPs was filtered to construct the upper intercalated layer of the composite film. The dispersion state of each component before filtration was investigated by Zeta potential analysis (Fig. S3). The Zeta potential of the deionized water dispersion of HLNPs was − 88.2 mV. When HLNPs were dispersed in water, phenolic hydroxyl and carboxyl groups provided surface charges to the nanospheres and promoted the formation of electrical double layers, which stabilized the dispersion of the nanospheres through the electrostatic repulsion force [41]. The Zeta potential of the deionized water dispersion of pure MXene was − 32.3 mV, while that of the MXene/HLNPs mixture was − 63.4 mV, suggesting that the incorporation of HLNPs enhanced the electrostatic repulsion between MXene sheets, leading to a more homogeneous colloidal dispersion in the system. The improved dispersion helped inhibit the re-stacking of MXene nanosheets and prevented the agglomeration in MXene/HLNPs dispersions [35, 42, 43]. This homogeneous and stable dispersion facilitated the formation of a homogeneous insertion structure of the membrane during the filtration process. The microscopic morphology of the MXene/HLNPs composite film prior to transfer to the carbon tape and the elastomer substrate was characterized in detail by TEM and SEM (Fig. 2). The images reveal the distinct underlying structure of stacked HLNPs and the upper layer of intercalated structure of MXene/HLNPs. The HLNPs layer increased the unevenness of the substrate and attenuated the parallel orientation of the MXene stack structure, providing smoother channels for ion transport. Moreover, HLNPs with the weak interface and the loose hollow structure acted as a protective phase to preserve the upper MXene layer. This architecture enabled slip and deformation during the subsequent stretch-release process of the wrinkled electrodes to improve the structural integrity of the electrode under tensile strain. As shown in Fig. 2c, d, the TEM and SEM images further revealed a significantly expanded pore structure and a reduced stacking density in the upper MXene/HLNPs intercalation structure of the composite film compared with the pure MXene film (Fig. 2g, h). Moreover, the aligned structure of MXene in the parallel direction was significantly weakened compared to the pure MXene film. The hierarchical architecture was further observed by SEM–EDS mapping of the cross section of the MXene/HLNPs film, as shown in Fig. 2k. Since lignin has a higher amount of carbon content compared to MXene, the uniform intercalation of HLNPs among the MXene layer can be clearly observed from the carbon signal in the EDS mapping images. Figure S4 shows the BET adsorption/desorption isotherms for MXene and MXene/HLNPs. The BET specific surface area of MXene was 23.4 m^2^ g^−1^, confirming the formation of lamellar Ti_3_C_2_. However, MXene/HLNPs demonstrated a significant increase in the specific surface area to around 92.5 m^2^ g^−1^, revealing that the insertion of nanospheres into MXene sheets substantially increased the surface area of the resultant material and the reduction of MXene stacking by HLNPs intercalation. This hollow lignin nanosphere micro-chamber structure was expected to provide more unobstructed ion-transport channels, thus enhancing the capacitive performance. In addition, the loose MXene arrangement provided more spatial redundancy for compression to help maintain the structural integrity of the film during the straining-releasing process.

The chemical characteristics of MXene/HLNPs films were further analyzed by FTIR, Raman spectroscopy, XRD, and XPS. As shown in Fig. S5, the FTIR spectra revealed that the peaks corresponding to the −OH stretching vibration shifted to lower wavenumbers and broadened upon the addition of HLNPs to MXene (from 3475 to 3416 cm^−1^). This shift indicated the increase of hydrogen bonded–OH, implying the formation of hydrogen bonds between HLNPs and MXene layers [44, 45]. The Raman spectra of pure MXene and MXene/HLNPs film are shown in Fig. S6. Both samples exhibited characteristic peaks of MXene at 270, 400, and 615 cm^−1^. Upon the addition of HLNPs, the two broad peaks at 1390 and 1560 cm^−1^, corresponding to the D and G peaks of amorphous and graphitic carbon, significantly increased. This confirmed the change in the MXene layer arrangement. Moreover, the XRD analysis demonstrated a shift of the (002) peak to lower angles in all MXene films containing HLNPs compared to pristine MXene. Consequently, the interlayer spacing distance could be calculated using Bragg’s law, which was found to be 1.14 nm for pristine MXene and 1.23 nm on average for all MXene/HLNPs films. These results indicated that the integration of HLNPs increased the interlayer spacing of MXene electrodes, which helped to effectively prevent restacking of MXene layers, and as a result, enhancing ionic accessibility and increasing the capacitances of MXene/HLNPs electrodes. X-ray photoelectron spectroscopy (XPS) was conducted to further analyze the bonding states in the pure MXene film and MXene/HLNPs film. The results are shown in Fig. S7. The C 1*s* and O 1*s* spectra indicated an increase in the percentage content of O=C–O and C–O bonds, as well as a decrease in C–Ti bonds in the MXene/HLNPs film compared to the pure MXene film. Additionally, a new C=O peak appeared in the MXene/HLNPs film. These changes were attributed to the incorporation of lignin components into the MXene layer [35].

The tensile tests were performed on the MXene films and MXene/HLNPs films. As shown in Fig. S10a, the MXene film exhibited a tensile strength of 6.9 MPa with an elongation at break of 1.4%. With the addition of HLNPs, the tensile strength increased to 7.1–7.8 MPa, and more notably, the elongation at break significantly improved (reached 2.2%–4.1%). As we have discussed before, this enhancement was due to the presence of bulges and microscopic nanosheet wrinkles caused by the hollow lignin nanospheres between the MXene layers, which firstly provided more free space for the stretching deformation of the MXene nanosheets (as seen in the SEM images of the fracture surface of MXene/HLNPs in Fig. 2). Secondly, the round nanospheres facilitated the sliding of the MXene nanosheet during stretching, allowing the film to withstand a greater deformation.

**Fig. 3:**
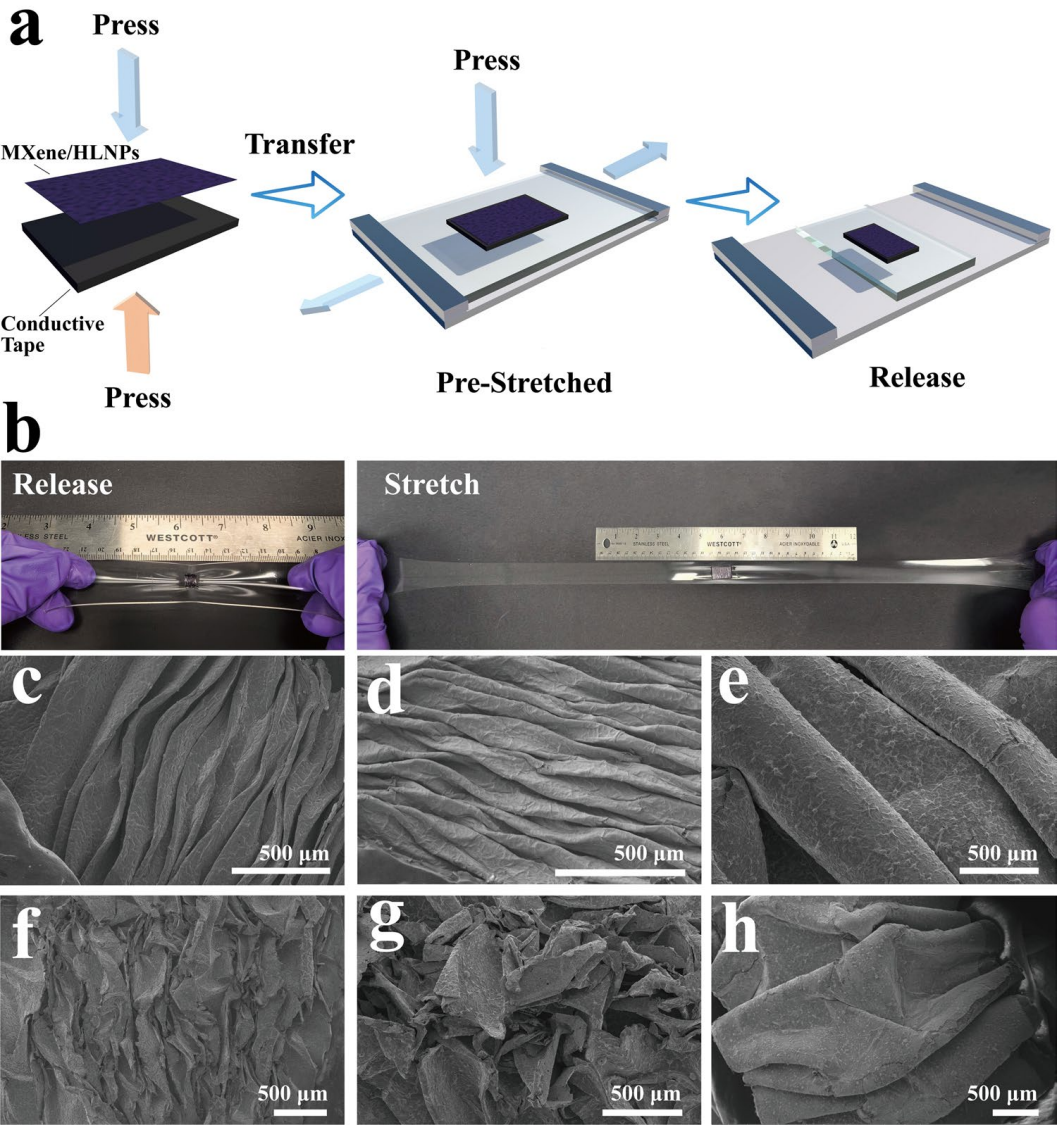
**a** Schematic diagram of stretchable electrode manufacturing procedure. **b** Illustration of stretchable electrodes in relaxed and stretched states. **c** Wrinkle surface of pure MXene film, and **d** MXene/HLNPs film transferred onto elastomer without conductive tape layer. **e** Wrinkle surface of MXene/HLNPs film transferred onto elastomer with conductive tape layer. **f** Pure MXene stretchable electrode, and **g** MXene/HLNPs stretchable electrode without conductive tape layer after 100 times stretch-release cycle at 600%. **h** MXene/HLNPs film stretchable electrode with conductive tape layer after 1000 times stretch-release cycle at 600%

Despite these improvements, the composite film remained susceptible to damage from large stretching. Hence, in this study, to achieve the fabrication of the highly stretchable electrode, a stretchable wrinkled structure of the MXene/HLNPs film was designed by taking advantage of the excellent stretchability of the VHB elastomeric substrate. Therefore, upon the complete drying, MXene/HLNPs composite film was transferred onto a carbon conductive tape (non-porous) with the HLNPs layer side down. This assembly was then laminated onto a uniaxially pre-stretched acrylic elastomer substrate pre-stretched at 600% strain. A slow and full relaxation of the elastomer substrate was conducted to complete the fabrication of the stretchable electrode. The fabrication procedure is illustrated in Fig. 3a. The stretchable electrodes in relaxed and stretched states are illustrated in Fig. 3b. The morphology of the wrinkled electrode was characterized using SEM (Fig. 3c–h) to investigate the structural integrity of flexible electrodes assembled by different procedures before and after tensile cyclic stretching. The results indicated that the presence of an intermediate conductive tape layer also played a crucial role in protecting the electrode structure during stretch cycling. For the wrinkled electrode attached to the conductive tape, after 1000 stress loading–unloading cycles at 600% strain, the originally highly oriented wrinkled structure tended to become looser, but there was no obvious fracture on the surface, indicating that the structural integrity of the electrode was well preserved (as shown in Fig. 3e, h). The cyclic tensile-release process of the flexible MXene/HLNPs electrodes after being stretched to 600% for 100 cycles on a universal tensile testing machine is shown in Video S1. The presence of the conductive tape layer led to a larger-scale and smoother wrinkles in the structure, which significantly reduced the compressive stress on the MXene film layer during the stretch-release processes. In contrast, for the wrinkled electrode without the protective conductive tape layer, the brittle nature of the MXene film made it difficult to withstand large-scale compressions, resulting in numerous cracks upon the first release of the pre-stretch (Fig. 3c, d). After just 100 stress loading–unloading cycles from 0 to 600% strain, the electrode surface exhibited severe fragmentation (Fig. 3f, g). In addition, by comparing the SEM images of the electrodes with and without HLNPs (including elastomer substrate and conductive tape layer) before and after 100 cycles of 600% cyclic tensile stretching, the incorporation of HLNPs effectively reduced the appearance of cracks (Fig. S8). This, together with the increase in material fracture strain with the incorporation of HLNPs observed in the mechanical tests (Fig. S10a), indicated that hollow lignin nanospheres provided more space for deformation and slip within the material, thus effectively protecting the structural integrity of the electrode. Additionally, SEM images revealed that the wrinkled structure of the electrode with HLNPs exhibited a high-density network of tiny vein-like wrinkles on top of the large-scale oriented wrinkles (see Figs. S8 and S9 for zoom-in view). This was attributed to the filling and supporting effect of HLNPs between the MXene layers during compression within the pre-stretch release. These vein-like tiny wrinkles also enlarged the interlayer spacing of the MXene layers, facilitating ion transport.

Furthermore, the mechanical properties of the integrated flexible electrode and each component were tested (Fig. S10). As shown in Fig. S10b, the excellent stretchability (reached 1405%) of the VHB substrate provided the elasticity to the electrode and defined the ultimate strain limit. Before approaching the set pre-strain (600%) of the electrodes, the stress–strain curves of the electrodes assembled with MXene film and MXene/HLNPs film were similar to that of the original VHB substrate, which indicated that the mechanical properties of the assembled electrodes within the set strain operating range (600%) were primarily provided by the VHB substrate. During stretching, the MXene composite film and the conductive tape experienced only the stretching and crumpling of the fold structure, without being subjected to large stresses, which helped protect the MXene composite film layer. This behavior was also verified by the similar cyclic tensile stress–strain curves at 0–600% strain for VHB and the integral electrode (Fig. S10c, f).

### Electrochemical Performance

To evaluate the electrochemical performance of the stretchable MXene/HLNPs electrodes, the cyclic voltammetry (CV), galvanostatic charge–discharge (GCD), and electrochemical impedance spectroscopy (EIS) experiments of the fabricated electrodes with and without protective layer of conductive tapes were tested in 1 M H_2_SO_4_ in a three-electrode system. Figure S11 shows the CV curves of the electrodes fabricated by directly transferring pure MXene film onto pre-stretched acrylic elastomer substrates without conductive tape layer. In the relaxation state, the MXene electrode exhibited an ideal double-layer capacitive behavior within the voltage window of − 0.2–0.4 V with the rectangular CV curves observed from 5 to 20 mV s^−1^. However, the electrode showed a low specific capacitance of 141 F g^−1^ (Calculated by GCD at 1 A g^−1^) and a highly distorted CV curve at an increased scan rate of 50 mV s^−1^, which was attributed to the significant resistance caused by cracking of the MXene film during the release process of the pre-stretched substrate. In addition, along with the increase in the stretching magnitude of the electrode between 0 and 600%, the CV curves at a scanning rate of 20 mV s^−1^ revealed significant degradation in the capacitive performance of the MXene stretchable electrodes without conductive tape layer, as shown in Fig. S12. This was also attributed to the significant fragmentation of the MXene layer during stretching. The EIS of MXene stretchable electrode without conductive tape layer at different tensile strains also verified the increase in resistance and decrease in capacitance performance during stretching (Fig. S13).

**Fig. 4:**
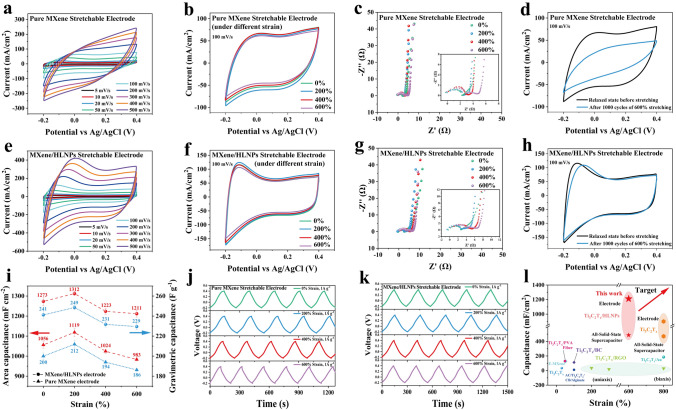
**a** CV curve of pure MXene stretchable electrode with conductive tape layer. **b** CV curve of pure MXene stretchable electrode with conductive tape layer under different strain from 0 to 600%. **c** EIS plot of pure MXene stretchable electrode under different strain from 0 to 600%. **d** CV curve of pure MXene stretchable electrode with conductive tape layer before and after 1000 cycles of 600% stretching. **e** CV curve of MXene/HLNPs stretchable electrode with conductive tape layer. **f** CV curve of MXene/HLNPs stretchable electrode with conductive tape layer under different strain from 0 to 600%. **g** EIS plot of MXene/HLNPs stretchable electrode under different strain from 0 to 600%. **h** CV curve of MXene/HLNPs stretchable electrode with conductive tape layer before and after 1000 cycles of 600% stretching. **i** Specific capacitance calculated by GCD test of pure MXene and MXene/HLNPs stretchable electrode under different strain from 0 to 600%. **j** GCD curves of the pure MXene stretchable electrode subjected to different strains. **k** GCD curves of the MXene/HLNPs stretchable electrode subjected to different strains. **l** Performance comparison of the MXene/HLNPs electrodes and supercapacitors with other reported MXene-based electrodes and supercapacitors

With the introduction of the conductive tape as a protective layer for the wrinkled structure of the MXene film, the electrochemical performance of the stretchable electrodes was significantly improved due to the strong mechanical properties of the conductive tape and its promotion of the conductive connectivity of the MXene film. As shown in Fig. 4a, the scan rate could be increased to 500 mV s^−1^ in the CV test. Correspondingly, a significant improvement in the specific capacitance of the electrode with the conductive tape layer was revealed compared to those without it (reached 200 F g^−1^ in the relaxation state, calculated by GCD at a current density of 1 A g^−1^, as shown in Fig. 4i, j). Furthermore, with the conductive tape layer protecting the structural integrity of the wrinkled MXene film during stretching and releasing, the electrode exhibited stable capacitive performance under different strains. As shown in Fig. 4b, the CV curves of the pure MXene stretchable electrode changed slightly under different strains from 0 to 600%. The EIS curves at different strains also verified the small resistance change and similar capacitive performance of the electrode during stretching (Fig. 4c). However, the capacitive performance of the electrode showed a significant decreasing trend as the number of stretch-release cycles increased, and the CV curves of pure MXene stretchable electrode with conductive tape layer before and after 1000 cycles of 600% stretching are shown in Fig. 4d. This was because the wrinkled structure constructed by the tightly stacked MXene layer was unable to withstand repeated bending and compression during numerous stretch-release cycles, resulting in a decrease in the structural integrity of the MXene layer.

The introduction of HLNPs to construct the hierarchical intercalation structure of MXene/HLNPs film further significantly increased the specific capacitance of the stretchable electrodes (reached 241 F g^−1^ which calculated by GCD at 1 A g^−1^, as shown in Fig. 4i, k), and the CV curves showed obvious pseudocapacitive behaviors and enhanced electrochemical kinetics (Fig. 4e). This improvement was partly attributed to the insertion of hollow lignin nanospheres that broke the originally highly oriented parallel stacking between the MXene sheets and enlarged the spacing of the MXene layers and the pore structure between the electrode films, which was demonstrated by SEM and XRD results. This disorientation in the parallel direction of MXene facilitated the construction of smoother ion transport channels. Moreover, the redox-active hydrazine/hydroquinone (Q/QH_2_) structure of lignin with high theoretical pseudocapacitance played an important role. In general, the high theoretical specific capacitance of lignin is difficult to exert due to its virtually electrically insulating nature. However, in this study, by inserting thin-walled single-pore hollow lignin nanospheres between the MXene layers, this composite material exhibited extremely high ion-accessible surface area. Furthermore, the intimate contact between the thin wall of lignin hollow sphere and the high electronic and ionic conductive MXene nanosheet also effectively promoted the realization of lignin’s pseudocapacitance. It is worth mentioning that, benefiting from the construction of the wrinkled electrode structure, the area specific capacitance of the electrodes was up to 1273 mF cm^−2^ (Calculated with the actual measured area in the relaxed state of the assembled electrodes), exceeding most currently reported stretchable electrodes, as shown in Fig. 4l (the detailed data comparison is presented in Table S1) [2, 19–21, 46–49]. Moreover, to investigate the effect of film thickness on its electrochemical properties, we tested the CV and GCD responses of the films with thicknesses of 2–10 μm, as shown in Fig. S14. It was found that in the thickness range of 2–10 μm, the thickness had no significant effect on the specific capacitance of the MXene/HLNPs film electrode. This also validated that the micro-chamber structure of hollow lignin nanospheres intercalated within the MXene layer provided more unobstructed ion-transport channels.

More importantly, the MXene/HLNPs stretchable electrodes exhibited almost strain-independent capacitive performance. The CV curves at 100 mV s^−1^ remained almost consistent across a wide range of tensile strains, ranging from 0% up to as high as 600%, as shown in Fig. 4f. The measured specific capacitance data of GCD showed minimal variation across different strain states, which coincided with the CV test, and further confirming the excellent electrochemical stability of the prepared stretchable electrodes (Fig. 4i, k). The capacitance of MXene/HLNPs stretchable electrode was not significantly affected even after 1000 cycles of 0–600% stretch-release (only 5% decrease, calculated by CV at 100 mV s^−1^, as shown in Fig. 4h). This outstanding electrochemical stability of the MXene/HLNPs composite electrodes under different strain values was attributed to the bottom-up hierarchical stacking structure in the MXene/HLNPs composite films and the protective effect of the conductive tape layer on the wrinkled electrode film. During stretching and the curling of the wrinkled structure after tensile release, the hollow lignin nanospheres dissipated the integrated stresses of the electrode film by slip and deformation between the MXene layers to improve the structural stability of the electrodes. The actual strain experienced by the active layers (MXene layers) of the wrinkled electrode during the stretching process was minimized. This observation was also verified by the minuscule resistance change and capacitance change observed from the EIS test of MXene/HLNPs composite electrode under different levels of strain (Fig. 4g).

### Stretchable All-Solid-State Supercapacitors

**Fig. 5:**
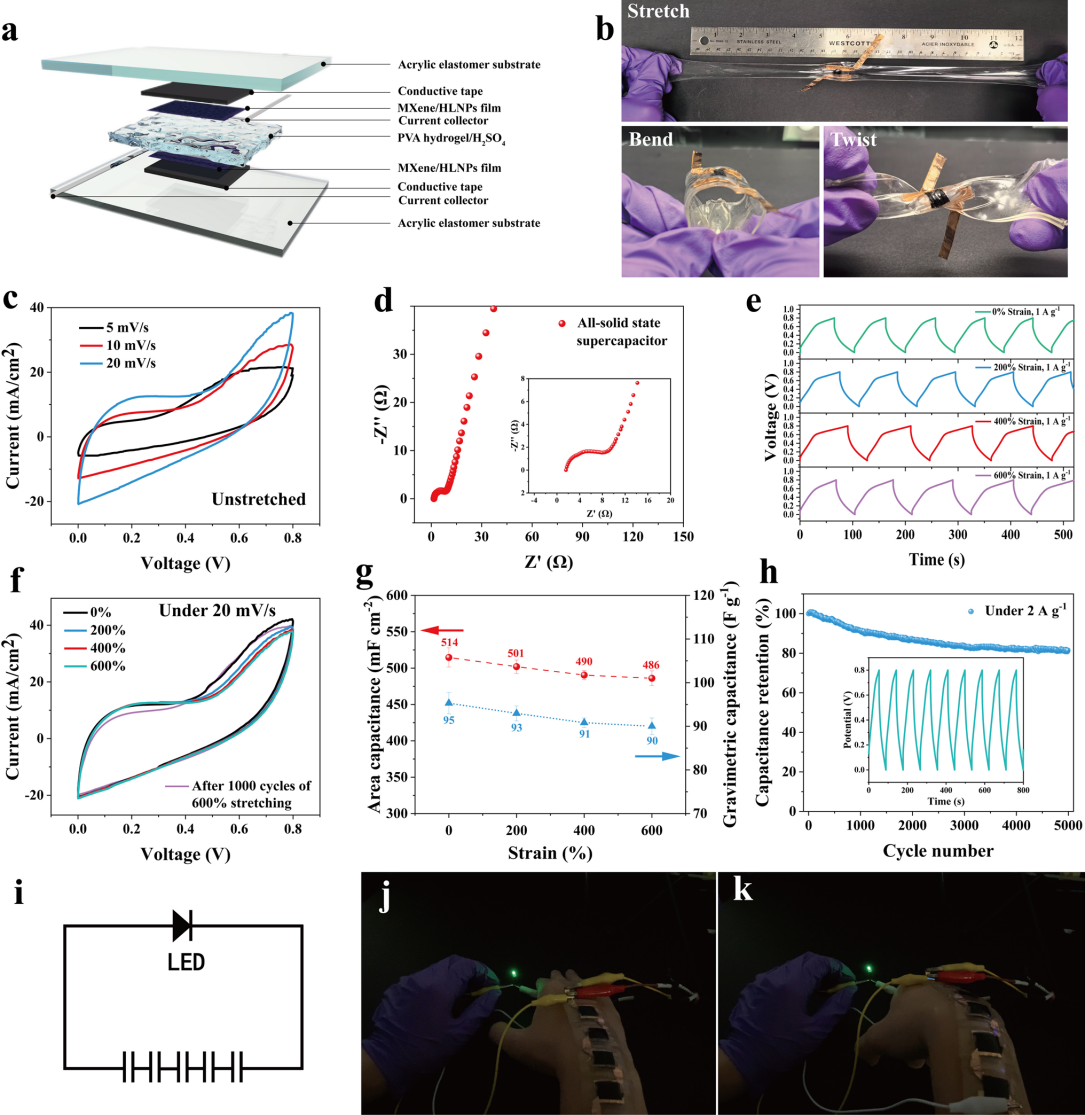
**a** Schematic diagram of the stretchable all-solid-state supercapacitor manufacturing procedure. **b** Illustration of the all-solid-state supercapacitor under stretching, bending, and twisting. **c** CV curve of the stretchable all-solid-state supercapacitor. **d** EIS plot of the stretchable all-solid-state supercapacitor. **e** GCD curves of the stretchable all-solid-state supercapacitor subjected to different strains. **f** CV curve of the stretchable all-solid-state supercapacitor under different strain from 0 to 600% and after 1000 cycles of 600% stretching. **g** Specific capacitance calculated by GCD at 1 A g^−1^. **h** Cycling stability of the stretchable all-solid-state supercapacitor for 5000 cycles under 2 A g^−1^. **i** Circuit of the fabricated patch with five serially connected supercapacitors. Schematic of the wearable supercapacitor-integrated device lighting up an LED in **j** a relaxation state and** k** a bending state

Given the excellent performance of stretchable electrodes, an all-solid-state stretchable supercapacitor was constructed using a pair of MXene/HLNPs electrodes and a PVA-H_2_SO_4_ hydrogel electrolyte layer, with the detailed structure of the supercapacitor shown in Fig. 5a. The CV curves of the symmetric supercapacitor exhibited capacitive behavior at scan rates ranging from 5 to 20 mV s^−1^ (Fig. 5c). However, as the scan rate increased, the shape of the CV curve became more resistive, indicating an increased influence of internal resistance in the electrode at high current densities. This was further confirmed by the higher resistance shown on the Nyquist plot (Fig. 5d) compared to the MXene/HLNPs electrodes before assembly. Despite a slight increase in the internal resistance of the system, the all-solid-state supercapacitor exhibited an area capacitance of up to 514 mF cm^−2^ (calculated based on GCD at 1 A g^−1^, as shown in Fig. 5e), owing to the design of the intercalation structure of the hollow single-pore lignin nanospheres between the MXene layers as described before and the full utilization of the pseudocapacitance of lignin. As stretchable supercapacitors, benefiting from the hierarchical stacking structure within the MXene/HLNPs composite film and the protective effect of the conductive tape layer on the wrinkled structure of the electrode film, the supercapacitor exhibited stable electrochemical performance under different strain levels. The ability of the stretchable supercapacitor to withstand stretching, bending, and twisting is shown in Figs. 5b and S15. Similar to the MXene/HLNPs electrodes before assembly, the CV curves (scanned at 20 mV s^−1^) of the supercapacitor were highly overlapped under different strain levels and after 1000 cycles of 600% stretch-release cycling (Fig. 5f). The gravimetric specific capacitance and area-specific capacitance of the all-solid-state capacitor under different strain levels were further tested by GCD at 1 A g^−1^, as shown in Fig. 5e, g. It was found that the capacitance of the capacitor only changed by 5.3% (from 514 to 486 mF cm^−2^) as the capacitor was stretched from 0 to 600%. Furthermore, to investigate the long-term performance of the all-solid-state supercapacitors, the capacitance retention of all-solid-state supercapacitors after 5000 charging and discharging cycles was tested at a current density of 2 A g^−1^ in the relaxation state, 600% stretching state, and after 1000 cycles of 0–600% cyclic stretching. The results showed that the supercapacitors maintained 82%, 87%, and 77% of their initial capacitance, respectively (as shown in Figs. 5h and S16). Additionally, the performance of the all-solid-state supercapacitors in different temperature and humidity environments was tested, as shown in Fig. S17 and Video S2. Compared to room temperature conditions, the supercapacitors exhibited some electrochemical degradation in cold environment at − 20 °C and in high-temperature dry environment at 50 °C. However, they still maintained an acceptable level of capacitance (reduced by 4.1% at 50 °C and by 24% at − 20 °C). This remarkable electrochemical stability under different strains and different environmental conditions as well as the cycling stability demonstrated a promising future for their wide application in various flexible electronics.

As the size and shape of MXene composite films were highly adjustable, the supercapacitors could be easily designed in various sizes and combinations, thus greatly expanding their practical application scenarios. Here, we designed a wearable patch of five serially connected supercapacitors as shown in Fig. 5i. Through this strategy we could extend the voltage range to drive a LED and even under bending deformation with various bending angles, as demonstrated in Fig. 5j, k, and Video S3.

## Conclusions

In summary, this work demonstrated a novel manufacturing approach for obtaining stretchable and high-pseudocapacitance supercapacitors through hierarchically intercalating single-pore hollow lignin nanospheres into MXene nanosheet layers. The construction of the gradient structure within the electrode enhanced the maintenance of its structural integrity during the stretch-release process. Thus, strain-independent capacitive performance of the electrode was achieved. Moreover, the intercalation of thin-walled single-pore hollow lignin nanospheres not only enlarged the interlayer spacing of MXene and increased the accessibility of ions, but also promoted the realization of lignin’s inherent pseudocapacitance. This innovative strategy endowed the stretchable electrodes with excellent pseudocapacitive behavior and enhanced specific capacitance (reached 1273 mF cm^−2^, 241 F g^−1^). The as-prepared assembled all-solid-state symmetric supercapacitors could withstand a uniaxial tensile strain up to 600% and demonstrated high specific capacitances of ∼514 mF cm^−2^ (95 F g^−1^). The 82% capacitance retention of the flexible supercapacitor after 5000 charge/discharge cycles and their electrochemical stability after 1000 times of 600% stretch-release cycling make these novel stretchable electrodes highly promising for a wide range of applications in various flexible electronic devices.

## Supplementary Information

Below is the link to the electronic supplementary material.

Supplementary file1 (DOCX 15357 KB)

Supplementary file2 (MP4 12391 KB)

Supplementary file3 (MP4 13768 KB)

Supplementary file4 (MP4 3826 KB)
