# Supplementary material for: Gradient-Layered MXene/Hollow Lignin Nanospheres Architecture Design for Flexible and Stretchable Supercapacitors
Source: Nanomicro Lett. 2024 Oct 17;17:43. doi: 10.1007/s40820-024-01512-3 (PMC11486903; doi:10.1007/s40820-024-01512-3)
Supplement: Supplementary file 1 — Supplementary file1 (DOCX 15357 KB) [file 40820_2024_1512_MOESM1_ESM.docx]

Supporting Information for

**Gradient Layered MXene/Hollow Lignin Nanospheres Architecture Design for Flexible and Stretchable Supercapacitors**

Haonan Zhang,^1, 2,^ **^‡^**, Cheng Hao^2,^ **^‡^**, Tongtong Fu^2^, Dian Yu^3^, Jane Howe^3^, Kaiwen Chen^4^, Ning Yan^2,^ *, Hao Ren^1,^ * and Huamin Zhai^1^

^1^ Jiangsu Provincial Key Lab of Sustainable Pulp and Paper Technology and Biomass Materials, NanJing Forestry University, NanJing, Jiangsu Province, 210037, P. R. China

^2^ Department of Chemical Engineering and Applied Chemistry, University of Toronto, 200 College Street, ON M5S 3E5, Canada

^3^ Department of Materials Science and Engineering, University of Toronto, 184 College Street, Toronto ON M5S 3E4, Canada

^4^ College of Materials Science and Engineering, Nanjing Forestry University, 210037 Nanjing, P. R. China

^‡^ Haonan Zhang and Cheng Hao contributed equally to this work.

*Corresponding authors. E-mail: [ning.yan@utoronto.ca](mailto:ning.yan@utoronto.ca) (Ning Yan); [renhao@njfu.edu.cn](mailto:renhao@njfu.edu.cn) (Hao Ren)

# Supplementary Figures and Tables


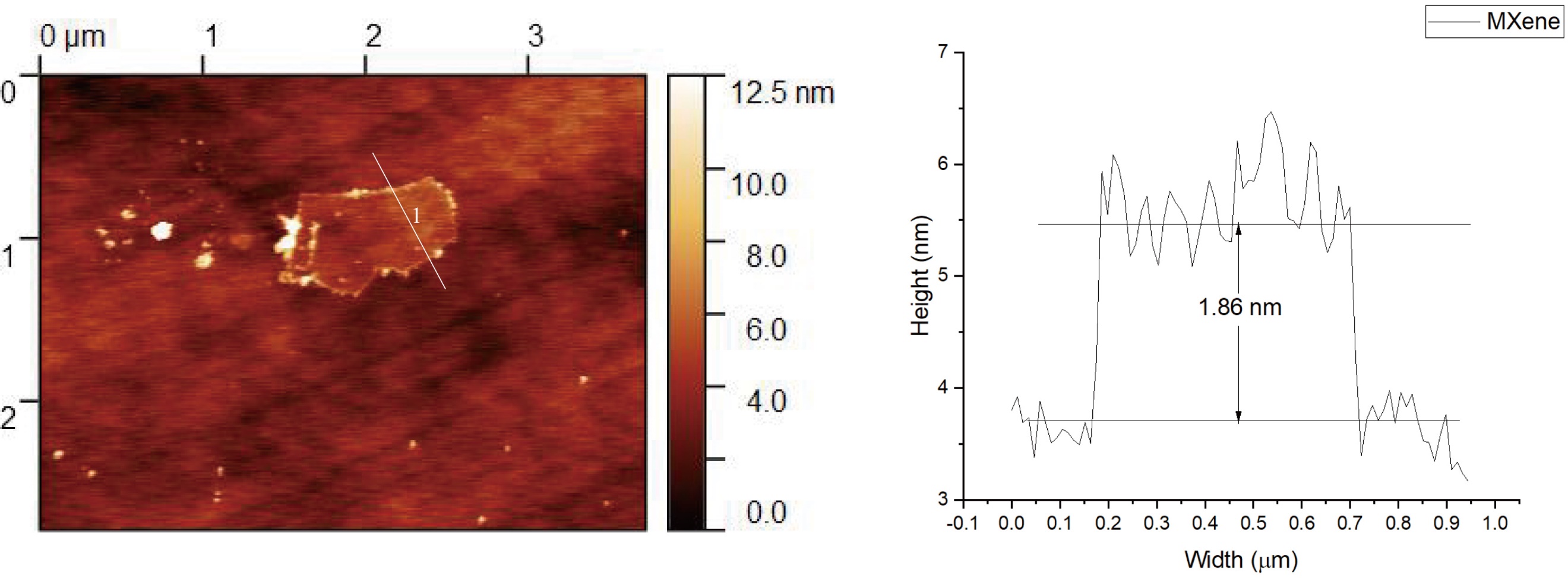


**Fig. S1** AFM image of the Ti_3_C_2_T*_x_* MXene nanosheet and thickness profile of the Ti_3_C_2_T*_x_* MXene nanosheet measured by line scan


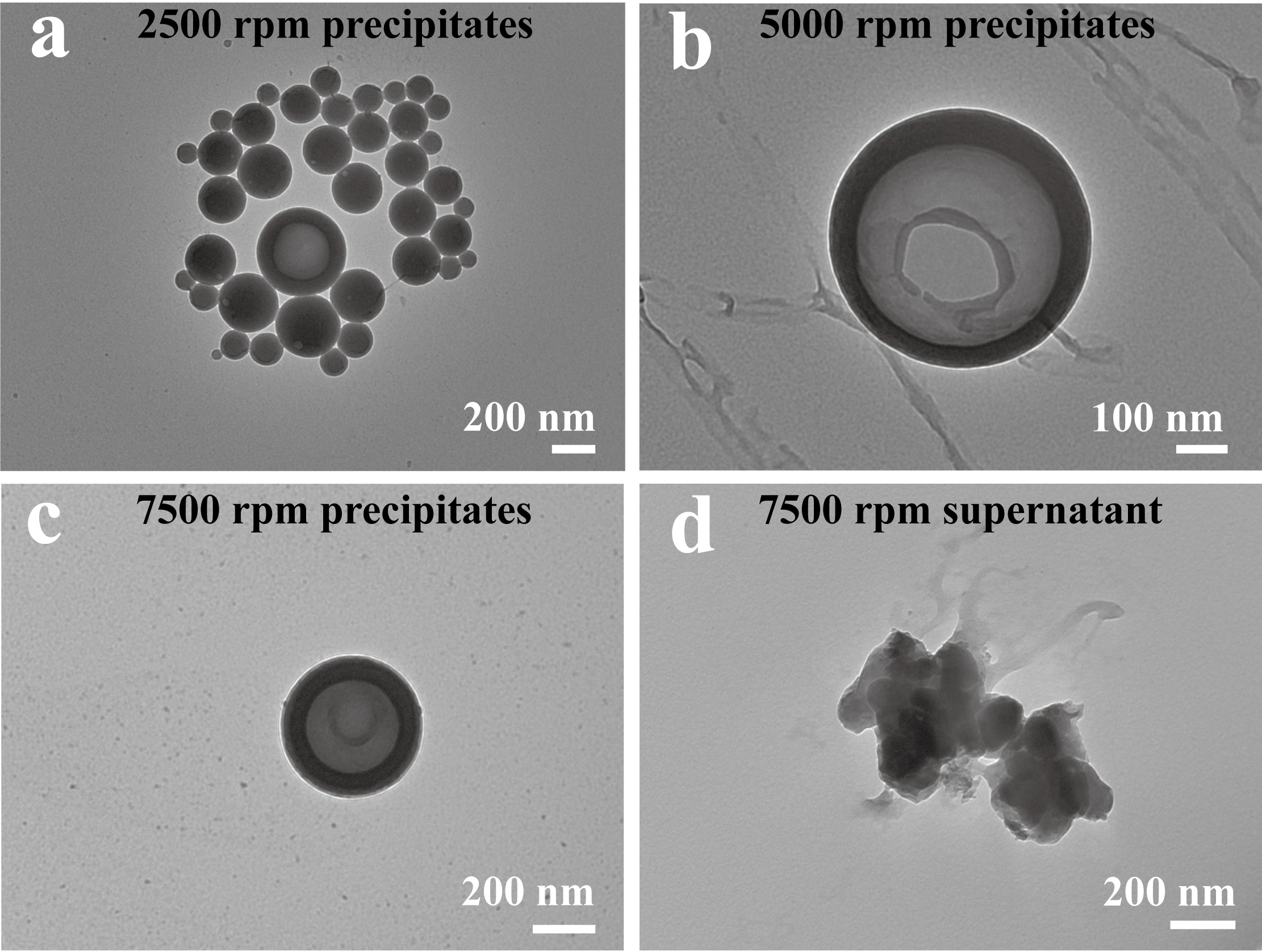


**Fig. S2** HLNPs obtained by grading at different centrifugal speeds: **a**) 2500 rpm precipitates. **b**) 5000 rpm precipitates. **c**) 7500 rpm precipitates. **d**) 7500 rpm supernatant. The relative centrifugal forces (RCF) for 2500 rpm, 5000 rpm, and 7500 rpm are 629 ×*g*, 2516 ×*g*, and 5660 ×*g*, respectively


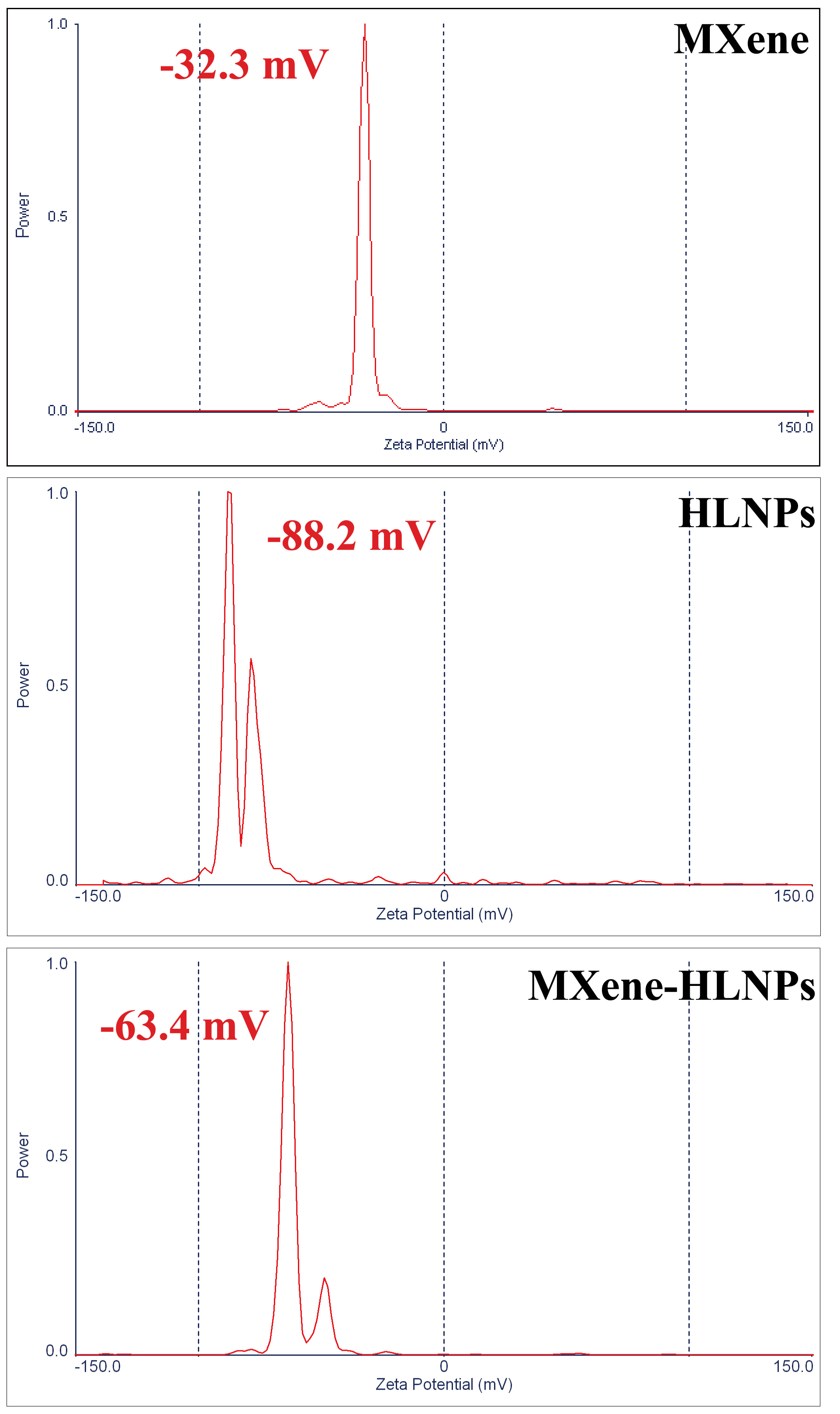


**Fig. S3** Zeta potential of the deionized water dispersion of HLNPs, MXene, and MXene/HLNPs mixture


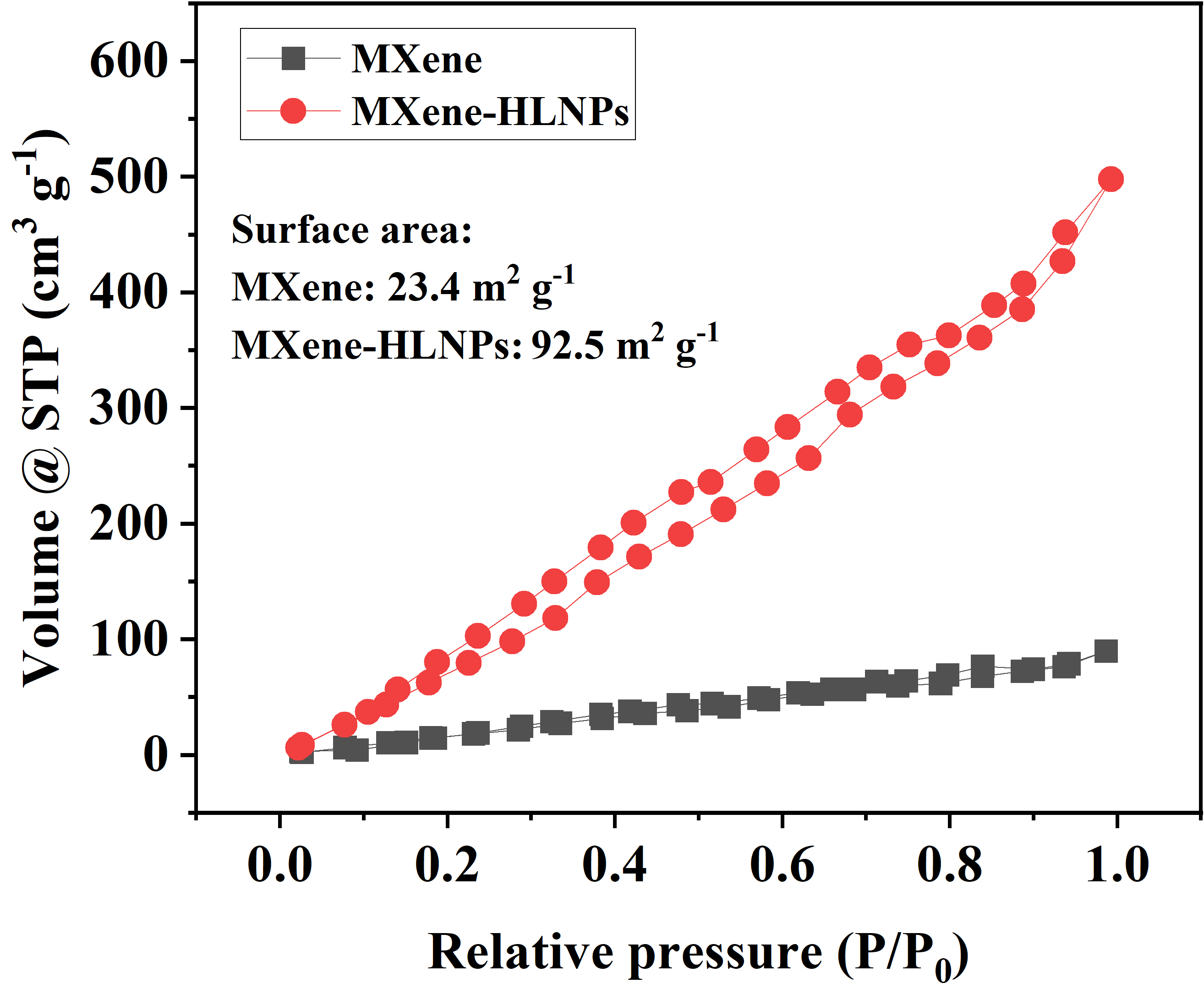


**Fig. S4** Nitrogen sorption isotherms and BET specific surface area of MXene film and MXene/HLNPs film


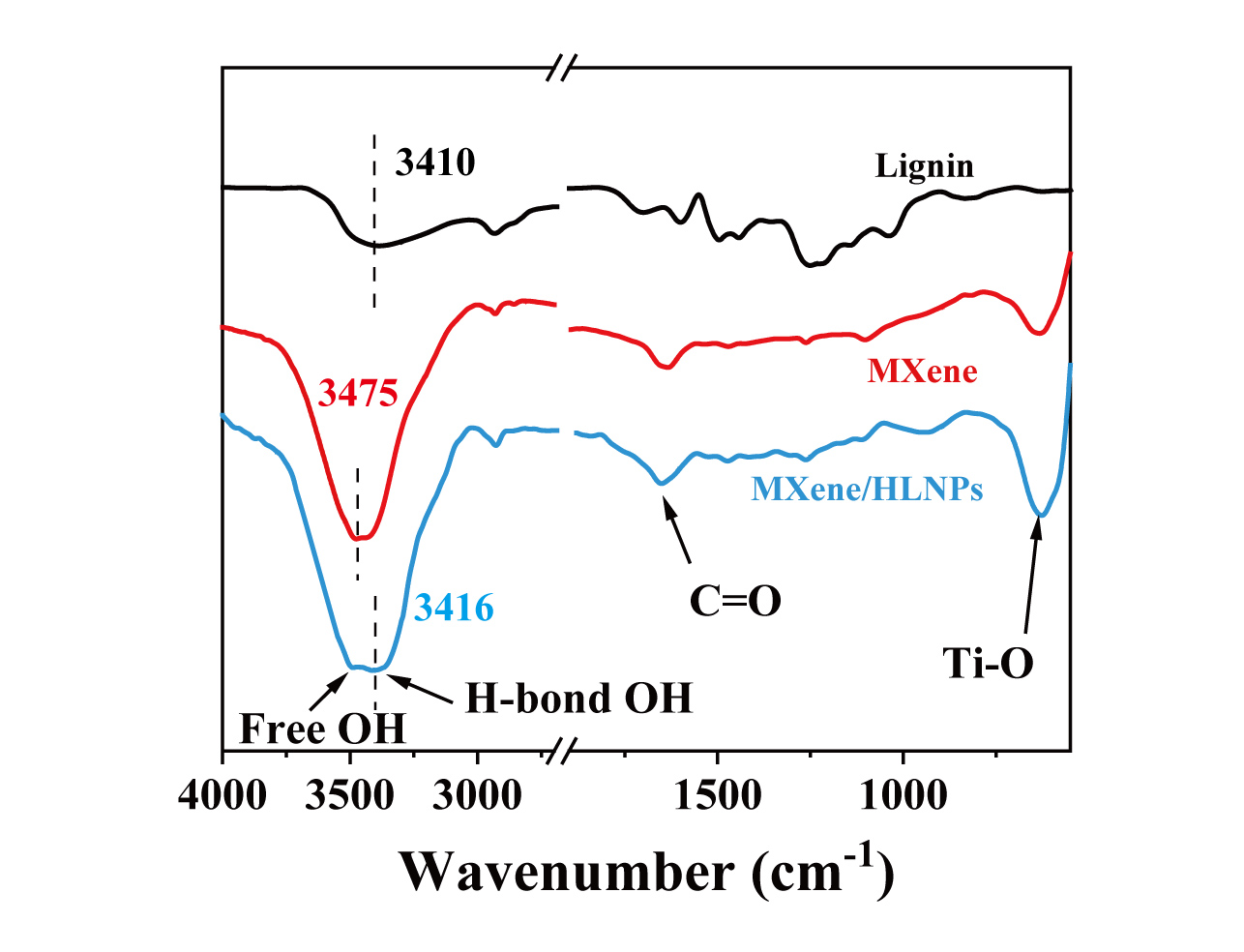


**Fig. S5** FTIR spectra of lignin, MXene film, and MXene/HLNPs film


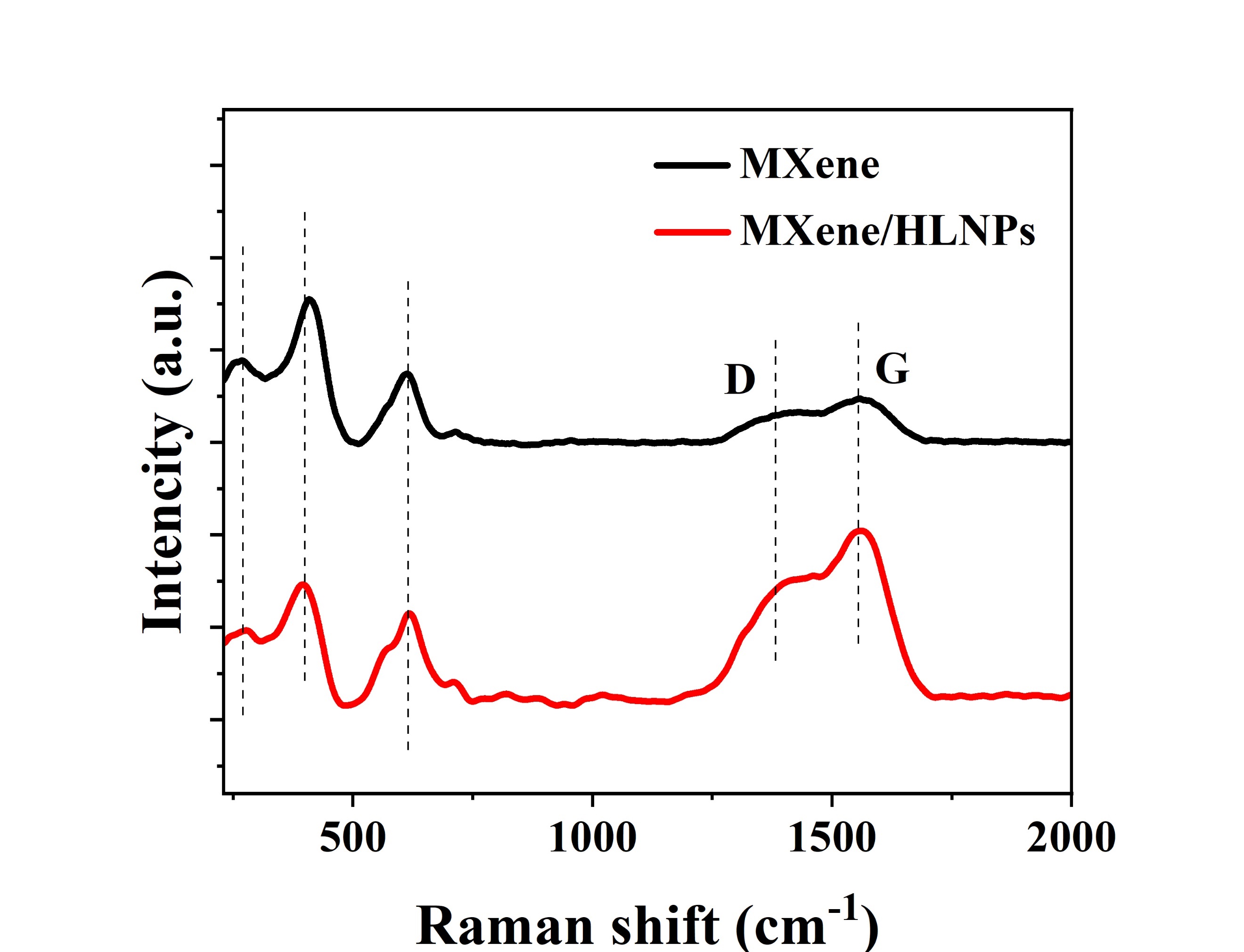


**Fig. S6** Raman spectra of MXene film, and MXene/HLNPs film


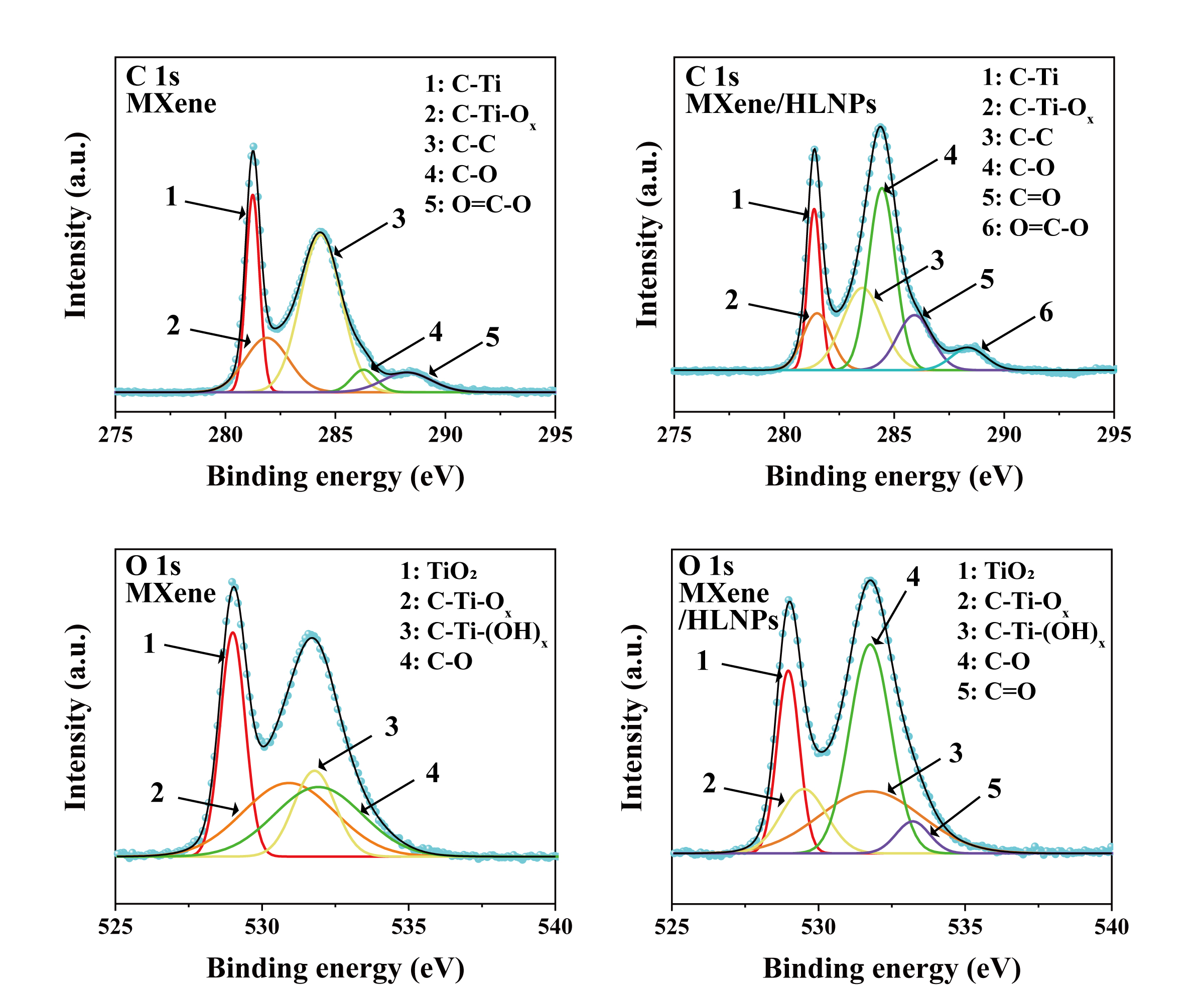


Fig. S7 XPS spectra of **a**) C 1s of pure MXene film, **b**) C 1s of MXene/HLNPs film, **c**) O 1s of pure MXene film, and **d**) O 1s of Xene/HLNPs film


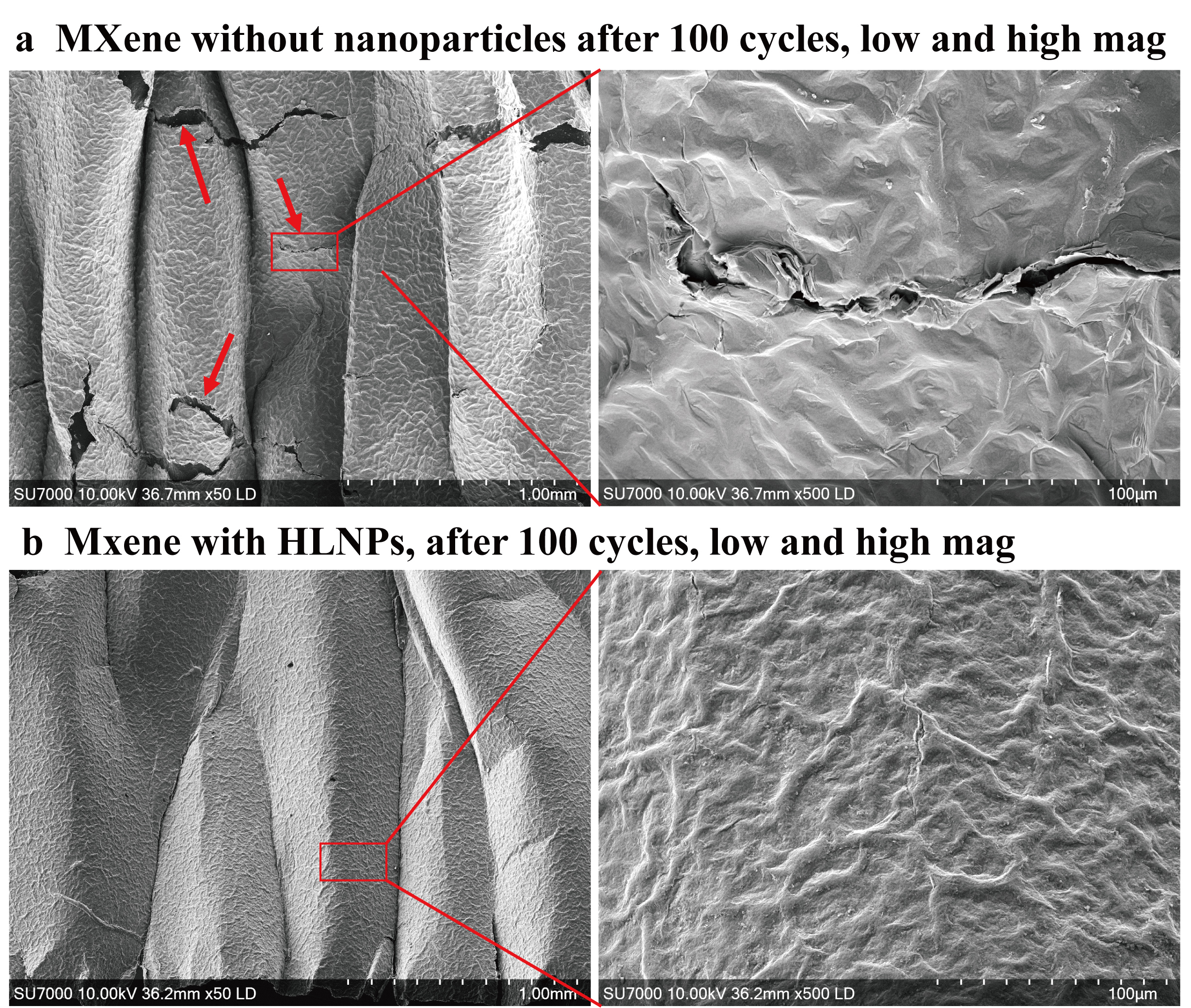


Fig. S8 MXene and MXene/HLNPs stretchable electrode after 100 times stretch-release cycle at 600%


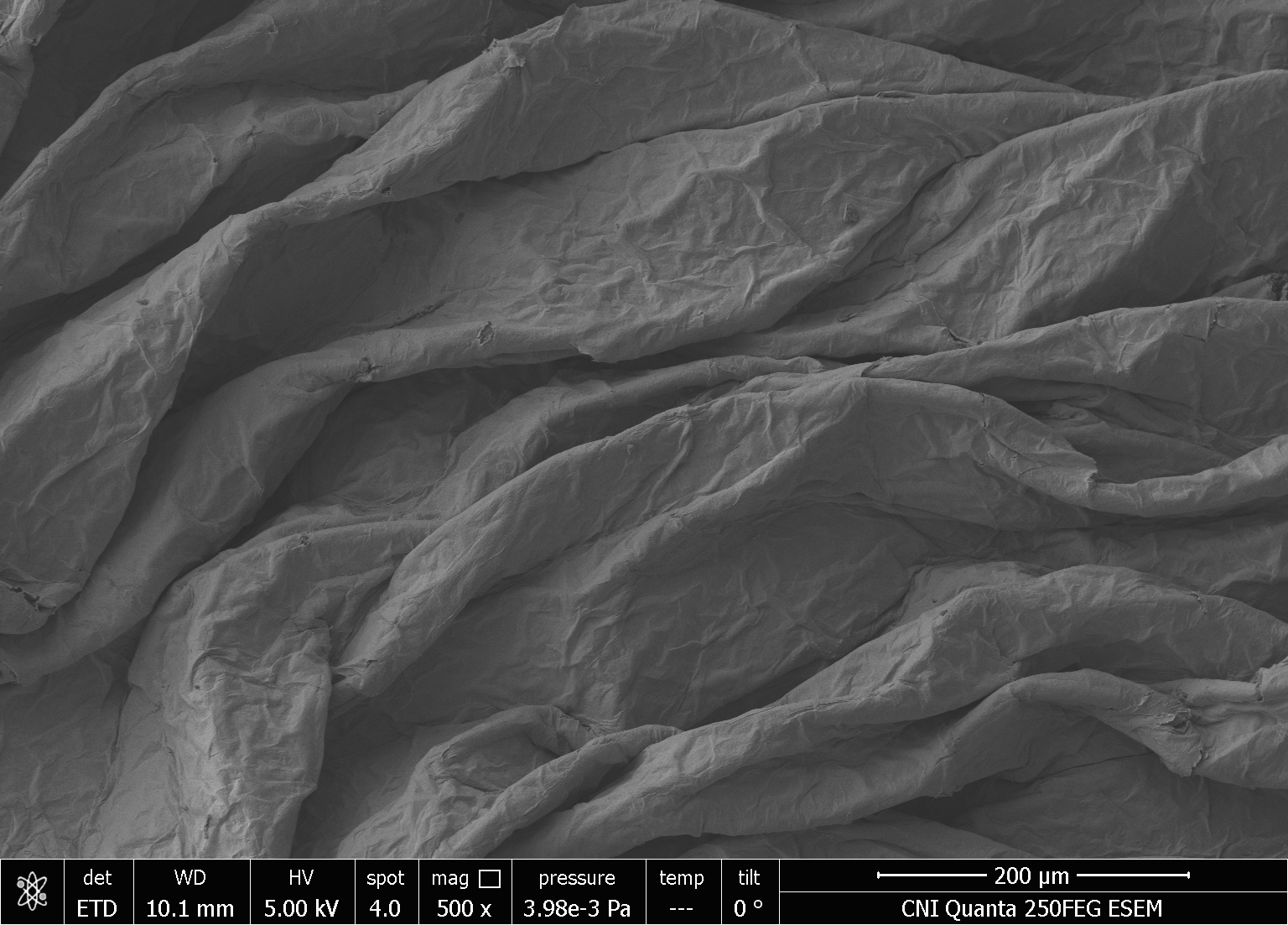


# **Fig. S9** Observation of the high-density network of tiny vein-like wrinkles on top of the large-scale oriented wrinkles in HLNPs added electrodes by SEM


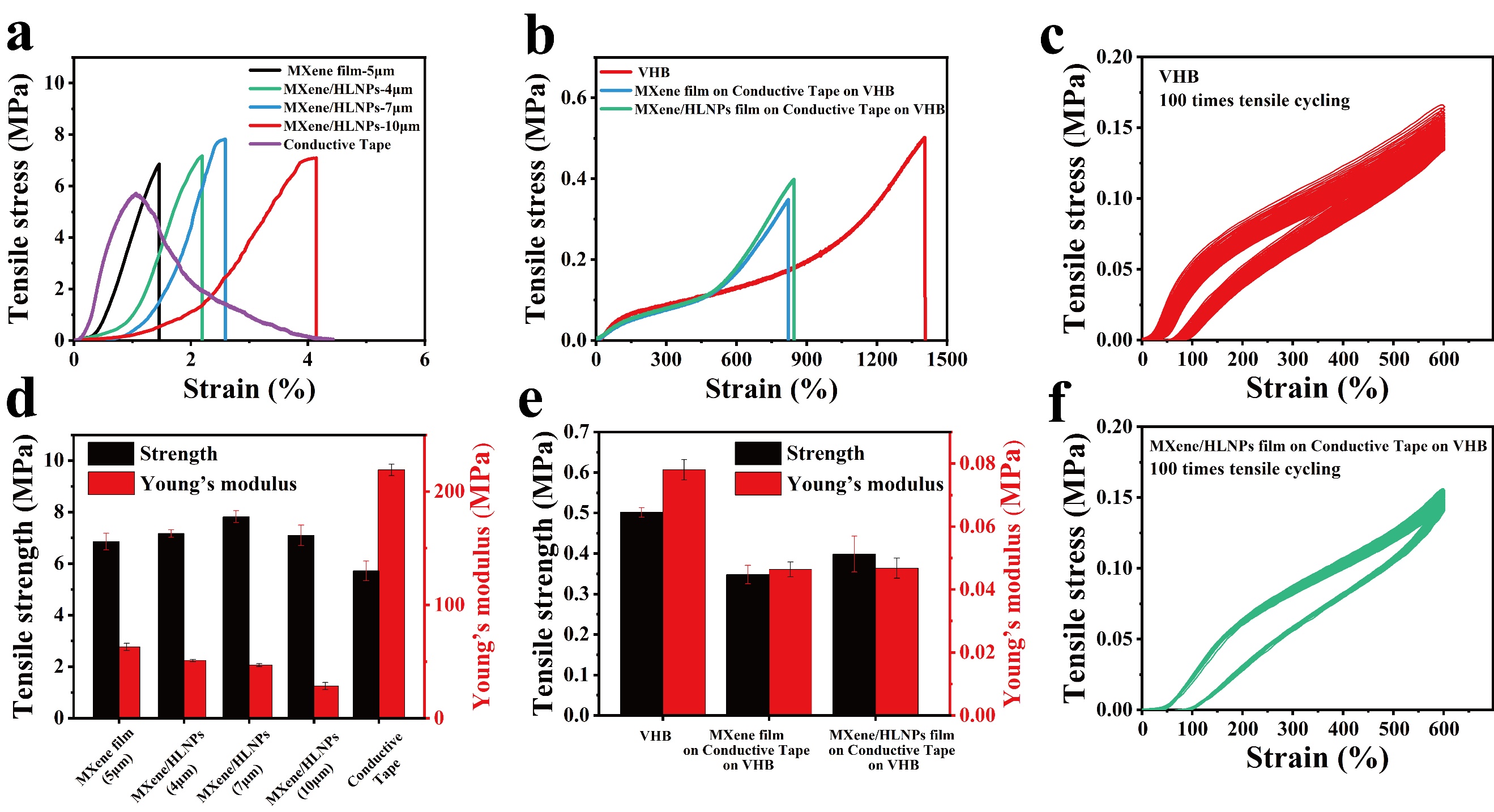


Fig. S10 a) Stress-strain curves of MXene film, MXene/HLNPs films with different thickness, and conductive tape. b) Stress-strain curves of VHB, MXene film on conductive tape on VHB and MXene/HLNPs film on conductive tape on VHB. c) Cyclic tensile test curves of VHB (100 times). d, e) tensile strength and young’s modulus of MXene film, MXene/HLNPs films with different thickness, conductive tape, VHB, MXene film on conductive tape on VHB and MXene/HLNPs film on conductive tape on VHB. f) Cyclic tensile test curves of MXene/HLNPs film on conductive tape on top of VHB (100 times)


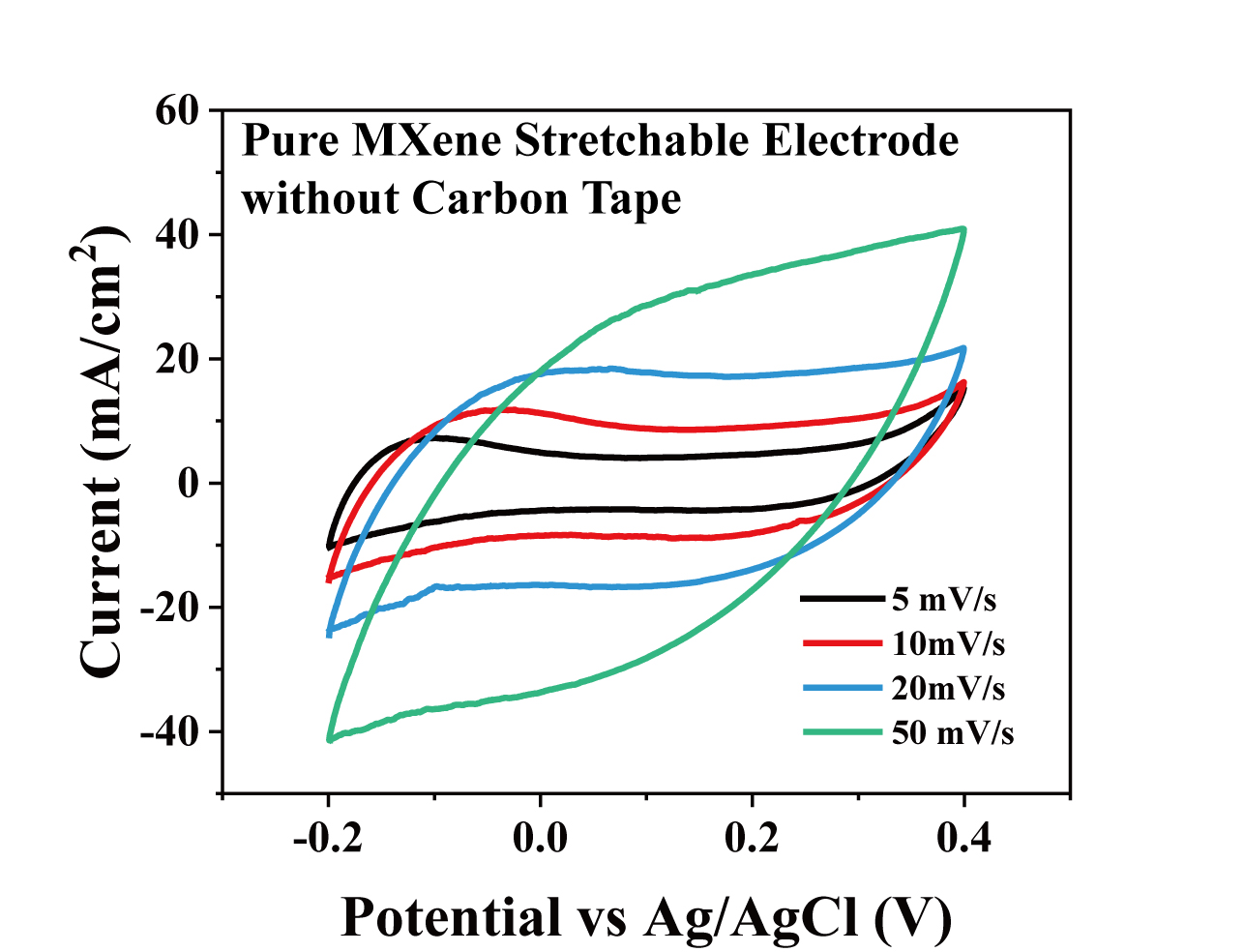


**Fig. S11** CV curve of pure MXene stretchable electrode without conductive tape layer


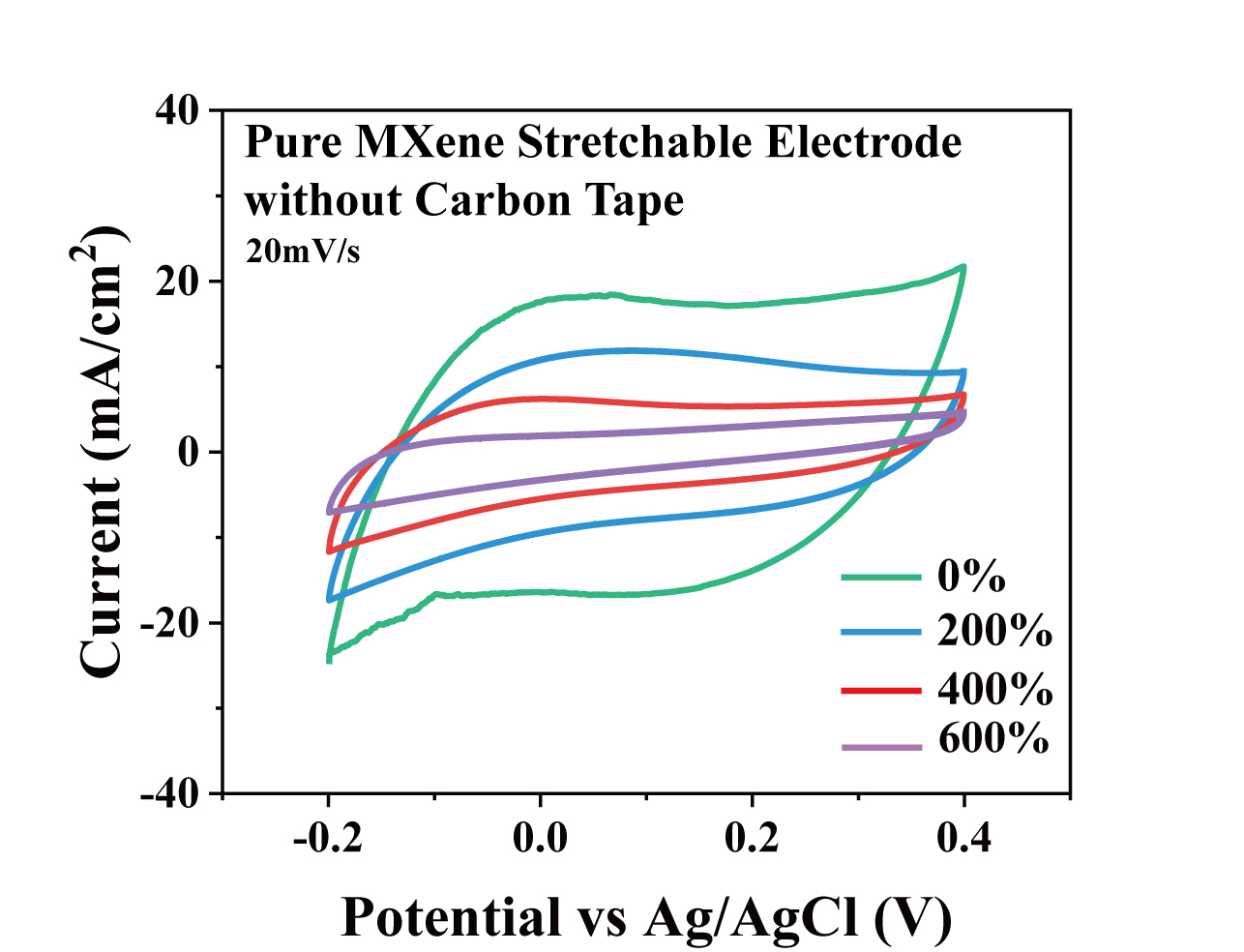


**Fig. S12** CV curve of pure MXene stretchable electrode (without conductive tape layer) at strain of 0%-600%


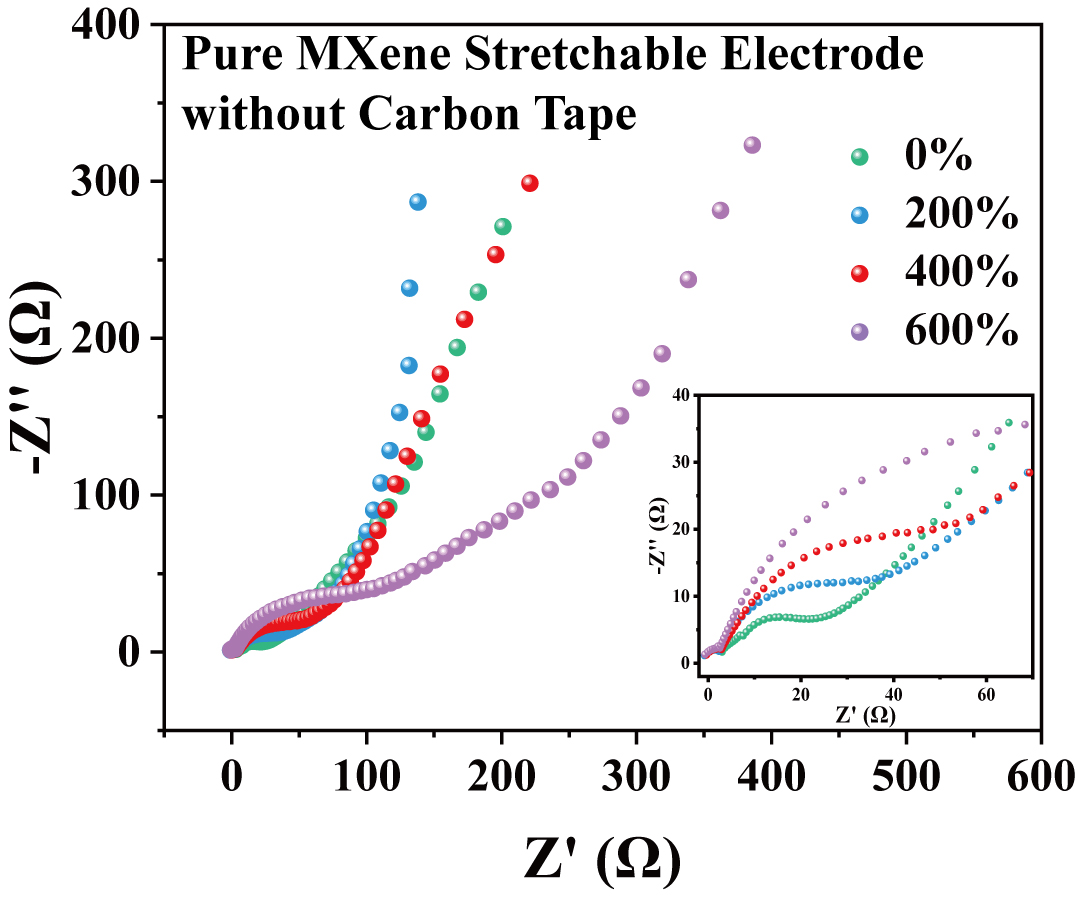


**Fig. S13** EIS of pure MXene stretchable electrode (without conductive tape layer) at strain of 0%-600%


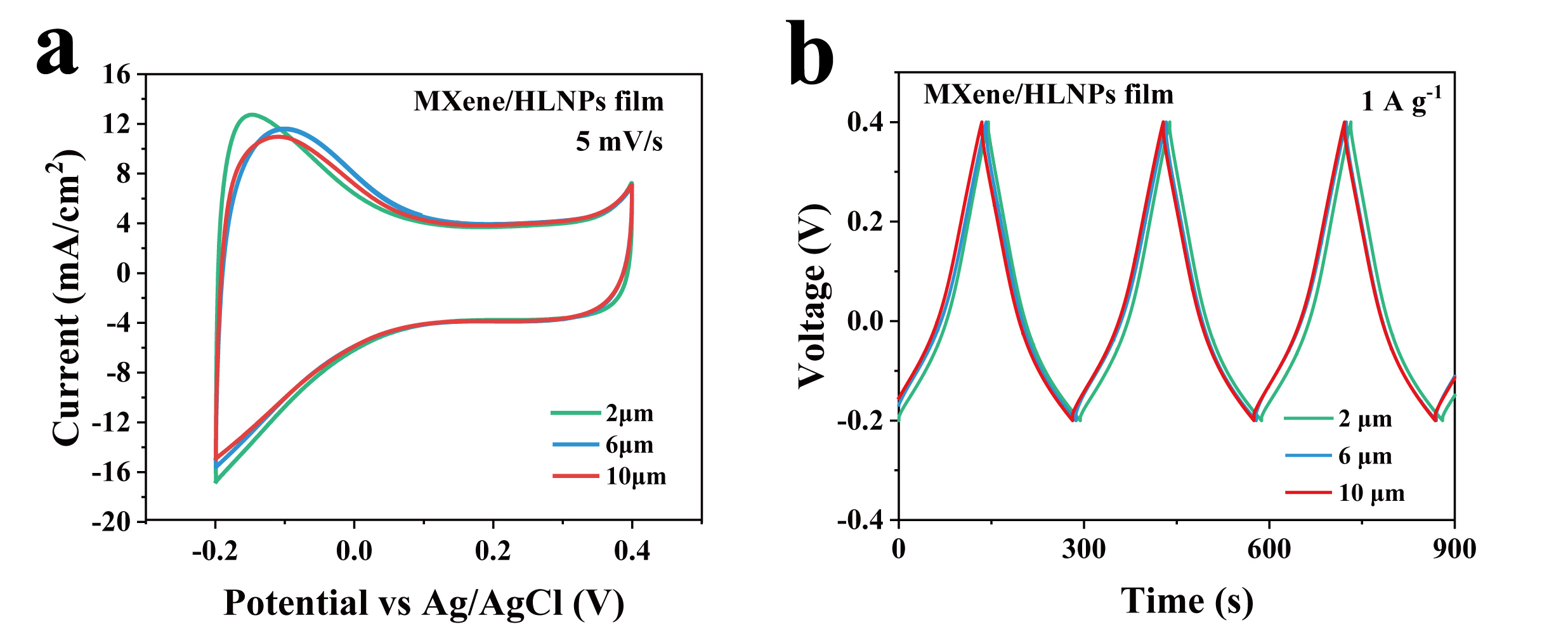


Fig. S14 a) CV curve of MXene/HLNPs stretchable electrode at different thicknesses. b) GCD curves of MXene/HLNPs stretchable electrode


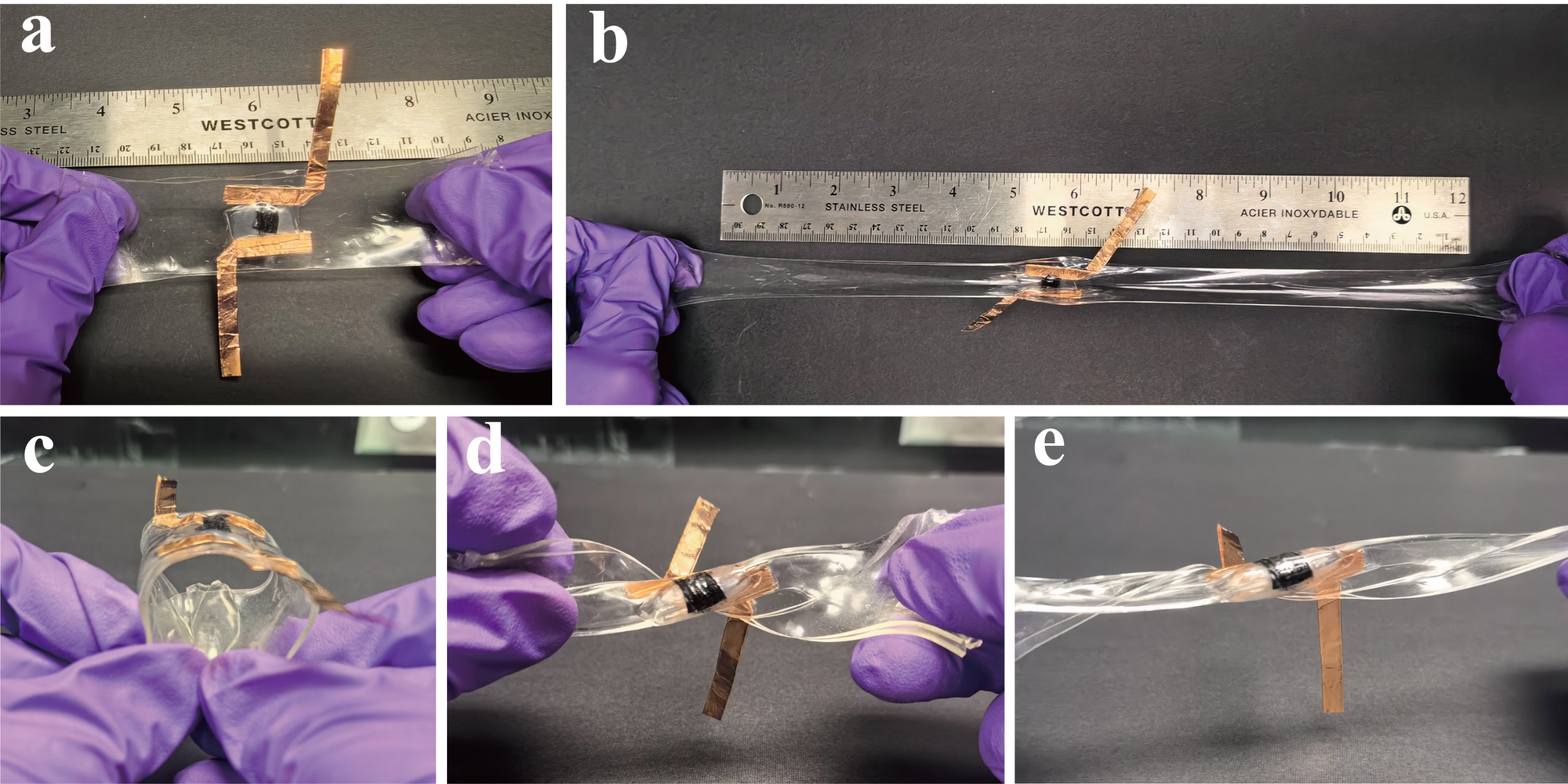


**Fig. S15** Illustration of the **a**) stretchable all-solid-state supercapacitor and its ability to withstand **b**) stretching, **c**) bending, **d**) twisting and stretch under twisting state


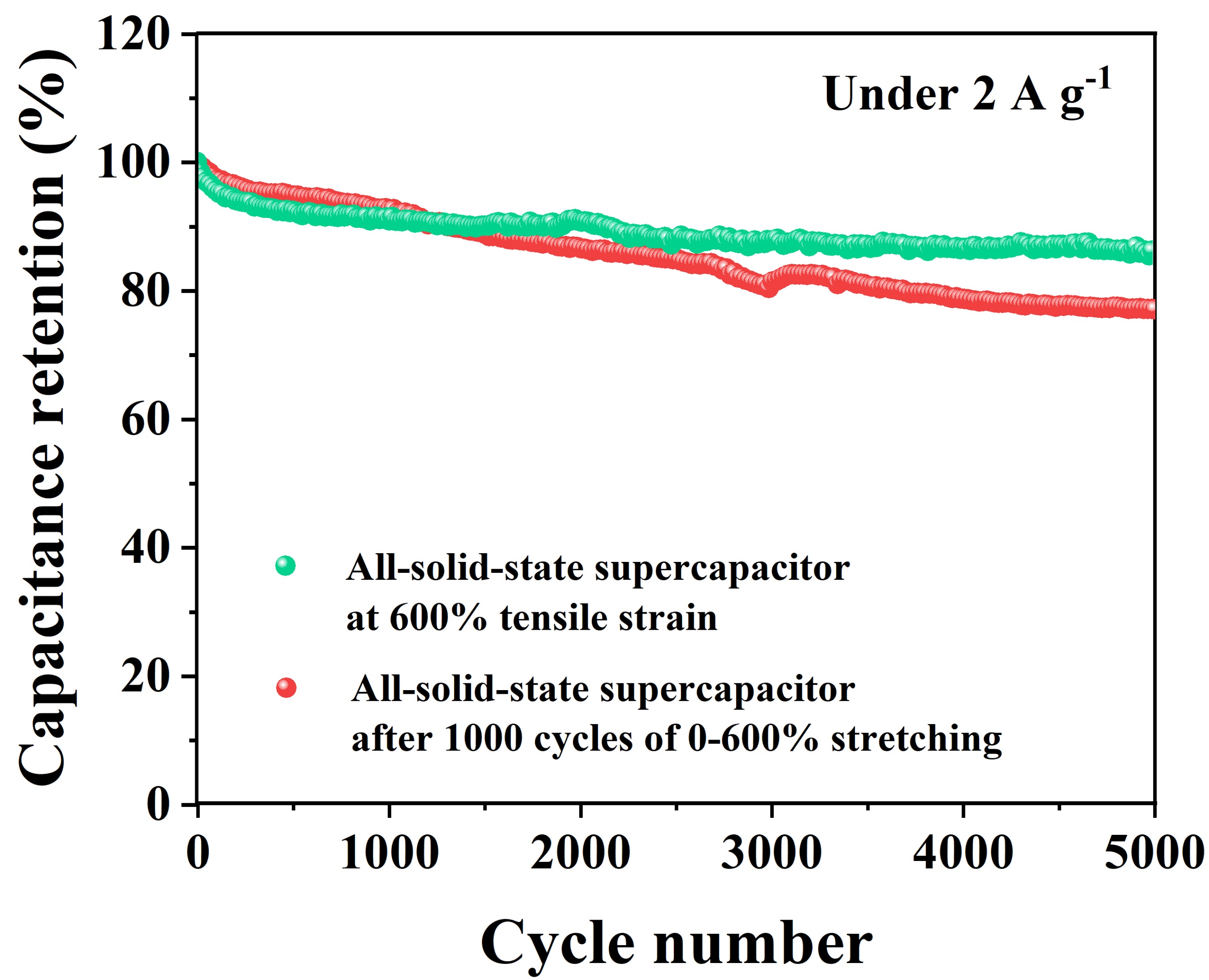


**Fig. S16** Cycling stability of the stretchable all-solid-state supercapacitor at 600% tensile strain and after 1000 cycles of 0-600% stretching for 5000 cycles under 2 A g^-1^


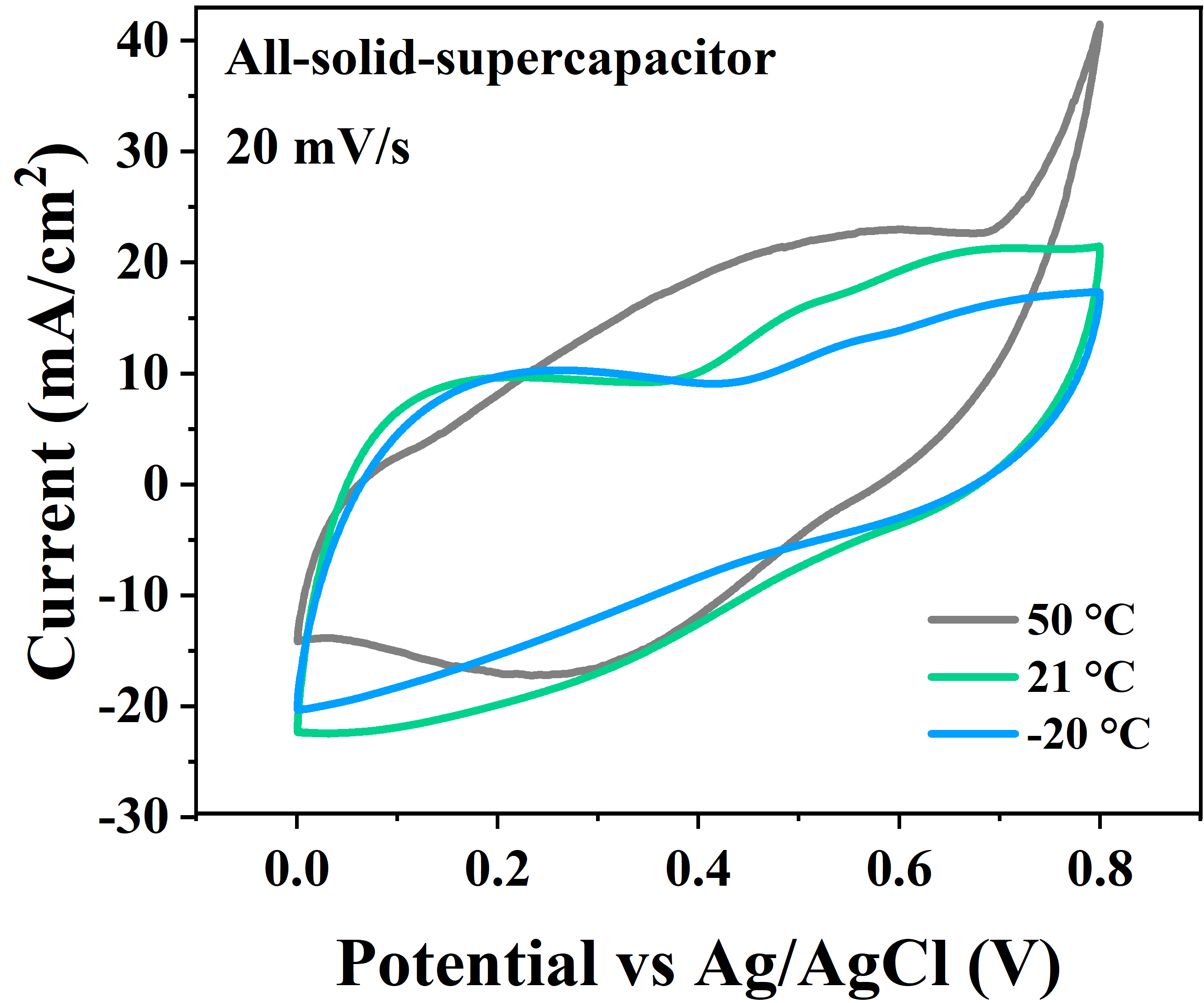


**Fig. S17** CV curve of the stretchable all-solid-state supercapacitor under different temperature

# Table S1 Comparison of the stretchable MXene/HLNPs electrode and all-solid-state supercapacitors with other MXene based electrodes and supercapacitors reported in literature

| **Electrode** | **Electrolyte** | **Stretchability** | **Cg (F/g)** | **CA**  **(mF/cm^2^)** | **Scan Rate** | **Ref.** |
| --- | --- | --- | --- | --- | --- | --- |
| Ti_3_C_2_T_x_/HLNPs | 1 M H_2_SO_4_ | 600% (uniaxis) | 241 | 1273 (~10 μm) | 1 A g^-1^ | This Work |
| Ti_3_C_2_T_x_ | 1 M H_2_SO_4_ | 800% (biaxis) | 64 | 470 | 2 mV s^-1^ | [S1] |
| Ti_3_C_2_T_x_/Au | 5 M H_3_PO_4_ | 800% (biaxis) |  | 185 | 2 mV s^-1^ | [S2] |
| Ti_3_C_2_T_x_/RGO | 1 M H_2_SO_4_ | 200% (uniaxis) | 115 | 35 | 1 A g^-1^ | [S3] |
| Ti_3_C_2_T_x_/RGO | 1 M H_2_SO_4_ | 800% (biaxis) | 93 | 29 | 1 A g^-1^ | [S3] |
| Ti_3_C_2_T_x_/RGO | 1 M H_2_SO_4_ | 300% (biaxis) |  | 19 | 0.1 A g^-1^ | [S3] |
| Ti_3_C_2_T_x_/BC | H_2_SO_4_ | 100% (uniaxis) |  | 111.5 (~13.3μm) | 0.75 mA cm^-2^ | [S4] |
| AC/Ti_3_C2T_x_/CB/Alginate | 1 M Li_2_SO4 | 100% (uniaxis) |  | 10.47 | 25 mV s^-1^ | [S5] |
| E-MXene | 1 M ZnSO_4_ | 50% (uniaxis) |  | 127.9 | 10 mV s^-1^ | [S6] |
| Ti_3_C_2_T_x_ | 1 M H_2_SO_4_ | 30% (uniaxis) |  | 33.3 | 10 mV s^-1^ | [S7] |
| Ti_3_C_2_T_x_/PVA fiber | H_3_PO_4_ | 48% (uniaxis) | 119.3 | 130.9 | 0.5 mA g^-1^ | [S8] |
| Ti_3_C_2_T_x_/graphene | 2 M KOH |  | 154 |  | 1 A g^-1^ | [S9] |
| Ti_3_C_2_T_x_/RGO | 1 M MgSO_4_ |  |  | 87 (~2 μm) | 2 mV s^-1^ | [S10] |
| Ti_3_C_2_T_x_/CNT | 1 M MgSO_4_ |  |  | 78 (~2 μm) | 2 mV s^-1^ | [S10] |

# Supplementary References

1. S. Feng, X. Wang, M. Wang, C. Bai, S. Cao et al., Crumpled MXene electrodes for ultrastretchable and high-area-capacitance supercapacitors. Nano Lett. **21**(18), 7561-7568 (2021). <https://doi.org/10.1021/acs.nanolett.1c02071>
2. J. Wang, Y. Qi, Y. Gui, C. Wang, Y. Wu et al., Ultrastretchable E-skin based on conductive hydrogel microfibers for wearable sensors. Small **20**(9), e2305951 (2024). <https://doi.org/10.1002/smll.202305951>
3. Y. Zhou, K. Maleski, B. Anasori, J. O. Thostenson, Y. Pang et al., Ti_3_C_2_T_x_ MXene-reduced graphene oxide composite electrodes for stretchable supercapacitors. ACS Nano **14**(3), 3576-3586 (2020). <https://doi.org/10.1021/acsnano.9b10066>
4. S. Jiao, A. Zhou, M. Wu, H. Hu, Kirigami patterning of MXene/bacterial cellulose composite paper for all-solid-state stretchable micro-supercapacitor arrays. Adv. Sci. (Weinh) **6**(12), 1900529 (2019). <https://doi.org/10.1002/advs.201900529>
5. T.-H. Chang, T. Zhang, H. Yang, K. Li, Y. Tian et al., Controlled crumpling of two-dimensional titanium carbide (MXene) for highly stretchable, bendable, efficient supercapacitors. ACS Nano **12**(8), 8048-8059 (2018). <https://doi.org/10.1021/acsnano.8b02908>
6. S. Li, T.-H. Chang, Y. Li, M. Ding, J. Yang et al., Stretchable Ti_3_C_2_T_x_ MXene microsupercapacitors with high areal capacitance and quasi-solid-state multivalent neutral electrolyte. J. Mater. Chem. A **9**(8), 4664-4672 (2021). <https://doi.org/10.1039/D0TA10560K>
7. L. Weng, F. Qi, Y. Min, The Ti_3_C_2_T_x_ MXene coated metal mesh electrodes for stretchable supercapacitors. Mater. Lett. **278**, 128235 (2020). https://doi.org/10.1016/j.matlet.2020.128235
8. W. Yu, Y. Li, B. Xin, Z. Lu, MXene/PVA Fiber-based Supercapacitor with Stretchability for Wearable Energy Storage. Fibers Polym. **23**(11), 2994-3001 (2022). <https://doi.org/10.1007/s12221-022-4389-4>
9. C. Zhao, Q. Wang, H. Zhang, S. Passerini, X. Qian, Two-Dimensional Titanium Carbide/RGO Composite for High-Performance Supercapacitors. ACS Appl. Mater. Interfaces **8**(24), 15661-15667 (2016). <https://doi.org/10.1021/acsami.6b04767>
10. M.-Q. Zhao, C. E. Ren, Z. Ling, M. R. Lukatskaya, C. Zhang et al., Flexible MXene/carbon nanotube composite paper with high volumetric capacitance. Adv. Mater. **27**(2), 339-345 (2015). https://doi.org/10.1002/adma.201404140
